# Comparative predictive value of immunotherapy biomarkers: a systematic review and network meta-analysis

**DOI:** 10.1097/JS9.0000000000004588

**Published:** 2026-01-21

**Authors:** Nuerye Tuerhong, Yang Yang, Junhao Feng, Benqi He, Peng Huang, Feng Wen, Qiu Li

**Affiliations:** a Department of Medical Oncology, Cancer Center, West China Hospital, Sichuan University, Sichuan, China; b West China Biomedical Big Data Center, Sichuan University, Sichuan, China; c Department of Clinical Medicine, The Second Hospital & Clinical Medical School, Lanzhou University, Lanzhou, Gansu Province, China; d Abdominal Oncology Ward, Division of Radiation Oncology, Cancer Center, West China Hospital, Sichuan University, Sichuan, China

**Keywords:** immunotherapy, predictive biomarkers, ctDNA, network meta-analysis, PD-L1, tumor mutational burden, precision oncology

## Abstract

**Background:**

Immunotherapy efficacy remains limited in over 60% of cancer patients, necessitating reliable predictive biomarkers. This network meta-analysis (NMA) compared the performance of 13 biomarkers to identify optimal predictors.

**Methods:**

We searched PubMed, OVID, Embase, Cochrane Trials, Web of Science, and trial registries (ClinicalTrials.gov, WHO ICTRP) from inception to 1 September 2025, for a comprehensive NMA evaluating 13 biomarkers (circulating tumor DNA [ctDNA], programmed cell death ligand 1 [PD-L1; at varying thresholds], tumor mutational burden [TMB], et al.). Subgroup analyses were performed for various cancers. Heterogeneity and publication bias were assessed.

**Results:**

This analysis included 54 634 patients from 194 clinical studies worldwide. ctDNA demonstrated the highest sensitivity (0.82, 95% CI: 0.72–0.89) and overall discriminative ability (DOR = 9.75, 95% CI: 5.20–16.73; AUC = 0.769). PD-L1 exhibited threshold-dependent performance: the ≥ 50% cutoff showed the highest specificity (0.78, 95% CI: 0.73–0.81) and diagnostic accuracy (DOR = 2.60, 95% CI: 1.86–3.52; AUC = 0.661) but the lowest sensitivity (0.42, 95% CI: 0.36–0.49), while the ≥ 1% cutoff achieved the highest sensitivity (0.68, 95% CI: 0.65–0.71) at the cost of the lowest specificity (0.48, 95% CI: 0.45–0.51). TMB provided a moderate balance of sensitivity (0.56, 95% CI: 0.50–0.60) and specificity (0.69, 95% CI: 0.65–0.73). MSI demonstrated the highest specificity overall (0.89, 95% CI: 0.85–0.93), but had limited sensitivity (0.36, 95% CI: 0.27–0.46). irAEs displayed relatively higher sensitivity (0.69, 95% CI: 0.60–0.77) with moderate specificity (0.59, 95% CI: 0.50–0.67). Among inflammatory markers, PLR (AUC = 0.623) showed slightly better predictive power than NLR (AUC = 0.613), while LIPI and LDH exhibited the least overall effectiveness (AUC = 0.585 and 0.544, respectively).

**Conclusion:**

Biomarker performance varies by cancer type and clinical context. ctDNA, PD-L1 (high thresholds, as ≥50%), and TMB are leading predictors, with combinations potentially optimizing performance. Future research must address heterogeneity and standardization to refine individualized immunotherapy strategies.

## Introduction

Cancer, which is the second most common cause of death in the world, is a persistent public health challenge for all countries^[1]^. Immunotherapy, which encompasses cytokine therapies, chimeric antigen receptor T-cell (CAR-T) immunotherapy, cancer vaccines, and immune checkpoint inhibitors (ICIs) such as programmed cell death 1 (PD-1), programmed cell death ligand 1 (PD-L1), and cytotoxic T lymphocyte-associated protein 4 (CTLA-4), etc., as a breakthrough therapy in the field of oncology, has significantly improved the clinical outcomes of various cancers^[2]^.

However, due to the individual variability in the efficacy of immunotherapy for solid tumors and the probability of progression patterns such as hyperprogression, identifying robust predictive biomarkers for immunotherapy response is essential for patient survival and prognosis^[3]^. The predictive markers presently utilized in clinical practice can be classified into several distinct categories. Among these, tumor-intrinsic features stand out, with microsatellite instability (MSI) status, tumor mutational burden (TMB), and liquid biopsy techniques that involve the analysis of circulating tumor DNA (ctDNA) serving as cornerstone biomarkers for patient selection across multiple malignancies. Furthermore, indicators associated with the tumor microenvironment also played a pivotal role, including the assessment of PD-1/PD-L1 and immune effector molecules, as tumor-infiltrating lymphocytes (TILs). Additionally, clinical and laboratory indicators such as the neutrophil–lymphocyte ratio (NLR) significantly contributed to the predictive process. Besides, increasing clinical studies suggested that the occurrence of immune-related adverse effects (irAEs) could be considered as a potential prognostic factor for predicting the efficacy of ICIs^[4–8]^.

It is notable that although markers such as PD-L1 expression, TMB, and MSI status have all been confirmed to have predictive value, traditional studies can only provide isolated effect size estimates and lack systematic comparisons of the predictive efficacy of different markers, thus failing to answer the key question of “Which marker has the greatest clinical guiding value.” The occurrence of evidence fragmentation can be traced back to the inherent constraints of traditional meta-analysis, which are capable of only facilitating pairwise direct comparisons. Consequently, the evaluation of efficacy across diverse markers is often confined to isolated, parallel realms, making it challenging to achieve a balanced and comprehensive assessment. Network meta-analysis (NMA) provides a methodological breakthrough for this purpose. By integrating the clinical research results of the efficacy of immunotherapy based on biomarkers, different detection methods were compared, and the overall ranking results were obtained to identify the biomarker-tumor type interaction^[9,10]^. This multidimensional analysis capability enables us to establish a “decision tree of immunotherapy predictive markers” based on evidence levels.

This comprehensive systematic review and network meta-analysis meticulously retrieved and examined clinical studies focusing on the predictive significance of relevant markers in the context of immunotherapy efficacy, and significantly contributed to advancing the paradigm shift in immunotherapy, moving away from a “blind trial” approach and toward a more precise and tailored treatment strategy.

## Methods

This NMA was conducted in adherence with PRISMA (preferred reporting items for systematic reviews and meta-analyses) and AMSTAR (assessing the methodological quality of systematic reviews) guidelines^[11,12]^, and the review protocol was registered on the International Prospective Register of Systematic Reviews (PROSPERO, No. CRD42023472497). Any revisions to the original registration information have been updated and made public on PROSPERO. This study also adheres to the reporting standards outlined in the TITAN (transparency in the reporting of artificial intelligence) Guideline Checklist for transparent research practices, and no AI was used in this study or manuscript preparation^[13]^. Supplemental Digital Content PRISMA Check List, available at: http://links.lww.com/JS9/G520

### Data sources & search strategy

The PubMed, OVID, Embase, Cochrane Trials, and Web of Science databases have been searched from inception to 1 September 2025, for relevant English-language literature. In addition, ClinicalTrials.gov and the World Health Organization International Clinical Trials Registry Platform (WHO ICTRP) were searched for completed clinical trials. We built a search query through the combination of the following subject words (including, but not limited to) and their free words: Neoplasm; Epidemiologic Study Characteristics; Immunotherapy; Immune Checkpoint Inhibitors; Microsatellite Instability; Programmed Cell Death 1 Receptor; CTLA-4 Antigen; Tumor mutational burden; Neutrophil-to-Lymphocyte Ratio; Treatment Outcome; Disease Progression; Predictive Value of Tests. The search strategy using the PubMed database as an example is detailed in Supplemental Digital Content Material 1, available at: http://links.lww.com/JS9/G519. Two researchers meticulously searched, compared, and integrated the data independently. A manual search of the reference lists and bibliographies of all included studies was conducted to identify any relevant articles not captured by the initial database search.

**HIGHLIGHTS**

- Circulating tumor DNA showed the highest sensitivity and discriminative power for predicting immunotherapy response.
- Programmed cell death ligand 1 demonstrated threshold-dependent performance. Higher cutoff values improved specificity at the cost of sensitivity.
- Biomarker performance is highly specific to both the cancer type and the treatment context, highlighting the need for predictive approaches that are tailored accordingly.
- Personalized combinations of biomarkers enhance overall predictive performance.
- Standardization and reducing heterogeneity remain key challenges in biomarker validation.
- Future research should refine biomarker selection to better identify immunotherapy-responsive patients.

### Eligibility criteria

This study strictly screened literature based on real-world data. The inclusion criteria required: (1) Patients with advanced solid tumors who received immunotherapy (including immune checkpoint inhibitors, CAR-T cell therapy or cancer vaccines); (2) Prospective and retrospective study, clinical trial, randomized controlled trial, cohort study, and case-control studies; (3) Biomarker detection was conducted in the study (covering PD-L1 expression, TMB, MSI status, TILs density, ctDNA level, inflammation-related markers, irAEs, etc.), and complete efficacy evaluation [objective response rate (ORR)] and survival outcomes [overall survival (OS), progression-free survival (PFS)] was reported. Primarily, complete ORR data to calculate sensitivity and specificity were required. Hematologic malignancies and neoadjuvant or adjuvant therapy were excluded, as were studies lacking biomarker status and treatment outcome data.

### Data extraction & quality assessment

In the included studies, the essential data were also punctiliously extracted by two researchers working independently. Any discrepancies that arose were diligently resolved through thorough discussions. The following details were recorded for each trial: the name of the study, the clinical trial number, the first author, the year of publication, the type of study, the sample size, the specific tumor type, the baseline characteristics of the participants, including treatment lines and immunotherapy settings, the biomarker stratification employed, and the treatment outcomes, which encompassed objective response rate, overall survival, among others. The missing variable in overall survival is marked as “not reported.”

Two researchers independently used the QUADAS-2 tool (modified version) implemented in Review Manager 5.3 (Cochrane Collaboration) to assess the methodological quality of the included studies. This assessment examined four key domains: (1) patient selection, (2) index test, (3) reference standard, and (4) flow/timing, with each domain rated for both risk of bias and applicability concerns.

### Statistical analysis

Utilizing the available ORR-related data, we extracted the values for true positives (TP), false positives (FP), false negatives (FN), and true negatives (TN) for each study by employing a 2 × 2 table. The heterogeneity of each included biomarker pattern was evaluated through forest plots and I^2^ analysis. An I^2^ statistic with values over 50% indicated substantial heterogeneity, and the random-effects model was applied for data analysis. Meanwhile, a fixed-effects model was used, particularly for subgroups with three or fewer studies or where the I^2^ statistic was below 50%. Additionally, meta-regression analyses were performed based on the source of the cut-off values, treatment settings, and treatment lines to determine the sources of heterogeneity. The ANOVA model was applied for the network meta-analysis of diagnostic test accuracy (DTA-NMA) to assess the effectiveness of various markers in predicting immunotherapy outcomes. Based on the bidirectional ANOVA model, two independent binomial distributions were used to describe the true positive and true negative of markers in patients who benefited from immunotherapy and those who did not, while considering the correlation between sensitivity and specificity.

NMA was mainly performed with the R package “Rstan” (R version 4.4.1). The R code was shown in the Appendix (see Supplemental Digital Content Material 2, available at: http://links.lww.com/JS9/G519). To improve accuracy and compare diagnostic assays one by one, calculations were repeated 7 times (model_code = model, chains = 2, iterations = 10 000, warmup = 5000, thin = 5). Diagnostic performance metrics evaluated included sensitivity (the true positive rate, calculated as TP/[TP + FN]), specificity (the true negative rate, calculated as TN/[TN + FP]), and diagnostic odds ratio (DOR) (a single indicator of overall performance, derived from [TP*TN]/[FP*FN]). Superiority was calculated as a net score from pairwise comparisons within the network. The overall accuracy was summarized using summary receiver operating characteristic (SROC) curves and the corresponding area under the curve (AUC). The league tables were generated for relative comparison purposes. The network diagram was drawn using the network diagram package on Stata (version 17.0 MP-Parallel Edition). Deeks’ funnel plots were generated for the objective response rate to assess potential publication bias. Statistical significance was tested using Egger’s linear regression test (*P* < 0.10 considered indicative of bias).

## Results

### Study selection and characteristics of the included studies

**Figure 1.**
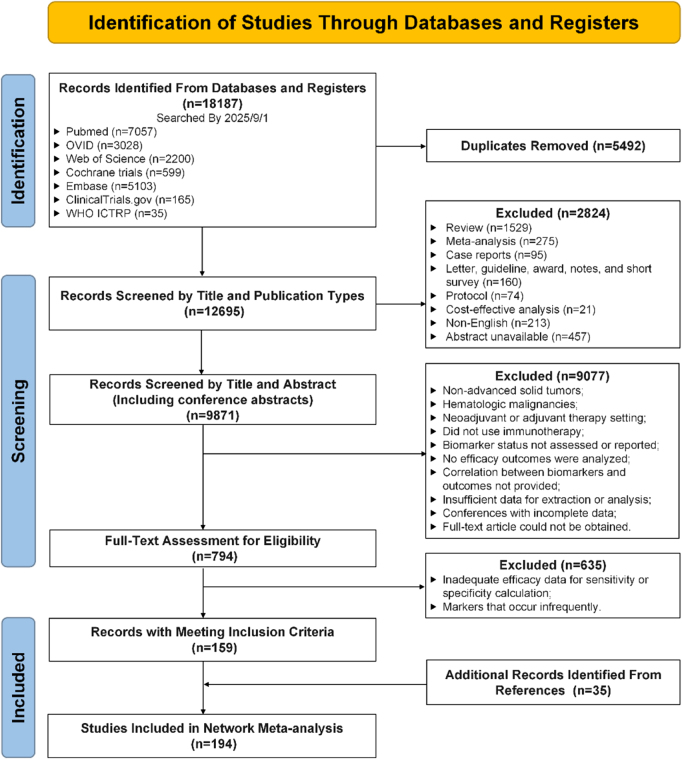
PRISMA flow diagram of study selection process. PRISMA (preferred reporting items for systematic reviews and meta-analyses) flow chart depicting the included studies for the meta-analysis addressing efficacy and biomarkers in pan-tumor patients receiving immunotherapy. Ultimately, 194 studies were included for network meta-analysis. WHO ICTRP: World Health Organization International Clinical Trials Registry Platform.

The initial systematic search retrieved a total of 17 987 records from five databases: PubMed (*n* = 7057), OVID (*n* = 3028), Web of Science (*n* = 2200), Cochrane Trials (*n* = 599), and Embase (*n* = 5103). Additionally, 200 records were obtained from clinical trial registries, including ClinicalTrials.gov (*n* = 165) and the World Health Organization International Clinical Trials Registry Platform (WHO ICTRP) (*n* = 35), up to 1 September 2025. After removing 5492 duplicates, 12 695 records underwent title and publication-type screening, excluding 2824 ineligible studies (reviews, meta-analyses, case reports, non-English articles, etc.). Subsequent abstract screening of 9871 records excluded 9077 studies based on predefined criteria. Full-text review of 794 articles further excluded 635 studies due to inadequate efficacy data for sensitivity/specificity calculation, or an insufficient number of rare biomarker literature. From the references of these 159 studies, 35 additional eligible publications were identified. Combined with the initial set, a total of 194 studies met all inclusion criteria and were incorporated into the network meta-analysis. The literature search and study selection flow are delineated in Figure 1.

**Figure 2.**
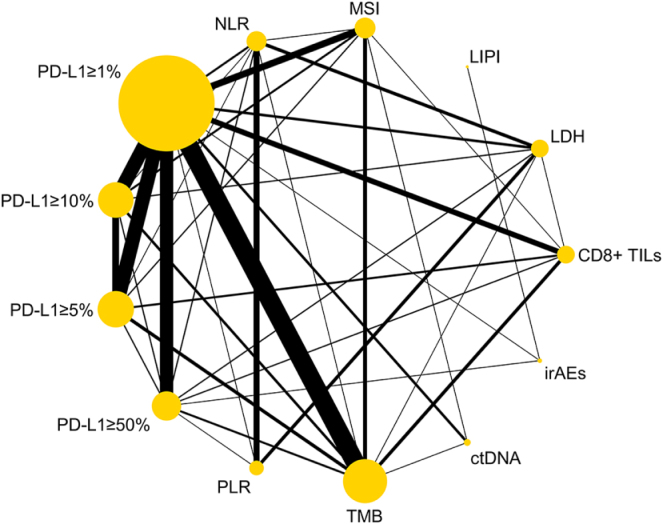
Network plot. The network diagram package in Stata (17.0 MP-Parallel Edition) was used to describe networks of the included trials that were directly comparable. Nodes and lines were weighted according to the number of studies involved for each treatment and direct comparison, respectively. ctDNA, circulating tumor DNA; CD8+ TILs, CD8-positive tumor-infiltrating lymphocytes; NLR, neutrophil-to-lymphocyte ratio; PD-L1, programmed death-ligand 1; PLR, platelet-to-lymphocyte ratio; LDH, lactate dehydrogenase; TMB, tumor mutational burden; MSI, microsatellite instability; irAEs, immune-related adverse events; LIPI, Lung Immune Prognostic Index.

This systematic analysis incorporated data from 194 clinical studies (2014–2025) involving a total of 54 634 patients across global research centers, with geographic representation from Asia (*n* = 95, 49%), the Americas (*n* = 65, 34%), Europe (*n* = 32, 16%), and Oceania (*n* = 2, 1%). The pooled dataset encompassed 392 biomarker assessments, demonstrating varied research focus across different predictive markers: PD-L1 expression analysis predominated [*n* = 212 across various cutoffs, particularly ≥1% (*n* = 110)], followed by genomic markers including tumor mutational burden (TMB, *n* = 68) and microsatellite instability (MSI, *n* = 14). Additional evaluations included CD8+ tumor-infiltrating lymphocytes (CD8+ TILs, *n* = 14), circulating biomarkers (ctDNA, *n* = 14), systemic inflammatory indices [NLR (*n* = 20), platelet–lymphocyte ratio (PLR) (*n* = 12), Lung Immune Prognostic Index (LIPI) (*n* = 11)], lactate dehydrogenase (LDH) [*n* = 10], and immune-related adverse events (irAEs, *n* = 17). Supplemental Digital Content Table S1.1–S1.5, available at: http://links.lww.com/JS9/G519provide an overview of the study population and biomarker distribution. About 28 different solid tumor types were analyzed, including lung cancer^[14–76]^, gastrointestinal (GI) cancer^[77–120]^, urogenital cancer^[19,121–153]^, head and neck cancer^[154–169]^, melanoma or skin cancer^[170–181]^, and other tumor types^[182–192]^. Lung cancer, particularly advanced non-small cell lung cancer (NSCLC), and gastrointestinal malignancies are the most widely studied categories. In addition, 14 studies were analyzed by the pan-tumor method^[193–206]^, and one study was a cancer of unknown primary site^[207]^. Complete study characteristics and methodological details are systematically presented in Supplemental Digital Content Table S2, available at: http://links.lww.com/JS9/G519. The mesh graph of available direct comparisons and the network of included trials are shown in Figure 2.

### Quality assessment

The quality assessment was conducted using the QUADAS-2 template, and the results are shown in Supplemental Digital Content Figure S1, available at: http://links.lww.com/JS9/G519. In terms of risk of bias, 55 retrospective studies were considered to have unclear risks in patient selection because they did not specify whether the patients were continuous or randomized. A total of 20.6% (40/194) of studies showed a high risk of bias in index test execution or interpretation. Reference standard bias risk was low across all studies. Only 12% (23/194) of studies were considered to have a high risk of bias in flow and timing because not all patients were included in the analysis. With applicability concerns, clinical applicability was generally good. About 6% (12/194) of studies had applicability limitations due to population specificity (such as specific tumor molecular subtypes, etc.).

### Network meta-analysis for comparing biomarkers in whole studies

A comprehensive network meta-analysis was conducted to assess the diagnostic performance of 13 biomarkers in predicting the response to immunotherapy across all the studies included. Several potential biomarkers were excluded from the network meta-analysis due to their rarity in the available studies, which prevented them from forming a connected network. The results of the NMA analysis are presented in Table 1. Key findings indicate a trade-off between sensitivity and specificity among various markers.

**Table 1:** Comparative predictive effects from network meta-analysis of all included studies.

Markers	Number of studies	Sensitivity	RANK	Specificity	RANK	DOR	RANK	Superiority	RANK	Relative Sensitivity	RANK	Relative Specificity	RANK
ctDNA	14	0.82 (0.72, 0.89)	1	0.67 (0.57, 0.76)	5	9.75 (5.2, 16.73)	1	16.1 (9, 21)	1	1.2 (1.06, 1.32)	1	1.39 (1.18, 1.62)	5
CD8+ TILs	14	0.69 (0.58, 0.79)	2	0.59 (0.49, 0.67)	7	3.41 (1.88, 5.78)	3	3.26 (0.2, 13)	2	1.02 (0.85, 1.18)	2	1.23 (1.02, 1.42)	7
irAEs	17	0.69 (0.6, 0.77)	3	0.59 (0.5, 0.67)	8	3.28 (2.19, 4.84)	4	3.01 (0.33, 11)	3	1.01 (0.88, 1.15)	3	1.22 (1.03, 1.41)	8
PD-L1 ≥ 1%	110	0.68 (0.65, 0.71)	4	0.48 (0.45, 0.51)	12	1.99 (1.71, 2.32)	10	0.57 (0.11, 1.67)	10	1 (1, 1)	4	1 (1, 1)	12
LDH	10	0.66 (0.55, 0.76)	5	0.4 (0.31, 0.49)	13	1.37 (0.77, 2.29)	13	0.18 (0.07, 1)	13	0.97 (0.81, 1.13)	5	0.84 (0.65, 1.04)	13
NLR	20	0.66 (0.57, 0.74)	6	0.49 (0.42, 0.56)	11	1.86 (1.24, 2.73)	11	0.44 (0.09, 1.67)	11	0.96 (0.83, 1.09)	6	1.02 (0.86, 1.19)	11
PLR	12	0.63 (0.51, 0.73)	7	0.57 (0.47, 0.66)	9	2.33 (1.26, 3.89)	9	1.11 (0.09, 5)	7	0.92 (0.74, 1.09)	7	1.19 (0.98, 1.39)	9
LIPI	11	0.57 (0.46, 0.69)	8	0.54 (0.44, 0.64)	10	1.65 (1.01, 2.54)	12	0.27 (0.07, 1.4)	12	0.84 (0.67, 1.02)	8	1.14 (0.91, 1.36)	10
TMB	68	0.56 (0.5, 0.6)	9	0.69 (0.65, 0.73)	4	2.82 (2.22, 3.52)	5	2.45 (0.33, 7)	4	0.82 (0.73, 0.89)	9	1.44 (1.34, 1.55)	4
PD-L1 ≥ 5%	45	0.54 (0.48, 0.6)	10	0.66 (0.62, 0.7)	6	2.34 (1.77, 3.03)	8	0.9 (0.14, 5)	9	0.79 (0.71, 0.88)	10	1.38 (1.27, 1.5)	6
PD-L1 ≥ 10%	32	0.44 (0.38, 0.51)	11	0.74 (0.7, 0.78)	3	2.34 (1.69, 3.11)	7	0.95 (0.2, 3)	8	0.65 (0.55, 0.75)	11	1.55 (1.44, 1.67)	3
PD-L1 ≥ 50%	25	0.42 (0.36, 0.49)	12	0.78 (0.73, 0.81)	2	2.6 (1.86, 3.52)	6	1.42 (0.33, 3)	6	0.62 (0.53, 0.72)	12	1.62 (1.5, 1.74)	2
MSI	14	0.36 (0.27, 0.46)	13	0.89 (0.85, 0.93)	1	4.93 (2.76, 8.02)	2	1.44 (1, 5)	5	0.53 (0.4, 0.67)	13	1.86 (1.74, 1.99)	1

Based on ORR-related data, TP, FP, FN, and TN values were extracted using 2 × 2 tables. Diagnostic accuracy network meta-analysis (DTA-NMA) was performed using an ANOVA model, implemented through the R package “Rstan” (10 000 iterations, number of chains 2, thinning 5), and sensitivity, specificity, DOR, and superiority were calculated. ctDNA, circulating tumor DNA; CD8 + TILs, CD8-positive tumor-infiltrating lymphocytes; DOR, diagnostic odds ratio; irAEs, immune-related adverse events; LDH, lactate dehydrogenase; MSI, microsatellite instability; NLR, neutrophil-to-lymphocyte ratio; PD-L1, programmed death-ligand 1; LIPI, Lung Immune Prognostic Index; PLR, platelet-to-lymphocyte ratio; TMB, tumor mutational burden.

Among these biomarkers, ctDNA exhibited the highest sensitivity (0.82, 95% CI: 0.72–0.89, rank 1), demonstrating superior ability to identify true responders, but with moderate specificity (0.67, 95% CI: 0.57–0.76, rank 5). CD8+ TILs followed with good sensitivity (0.69, 95% CI: 0.58–0.79, rank 2) but lower specificity (0.59, 95% CI: 0.49–0.67, rank 7). For PD-L1, higher cutoff values improved specificity at the cost of sensitivity. PD-L1 ≥ 50% exhibited the lowest sensitivity among PD-L1 thresholds (0.42, 95% CI: 0.36–0.49, rank 12) but achieved the second-highest specificity (0.78, 95% CI: 0.73–0.81, rank 2). PD-L1 ≥ 10% showed a sensitivity of 0.44 (95% CI: 0.38–0.51, rank 11) with a specificity of 0.74 (95% CI: 0.70–0.78, rank 3). PD-L1 ≥ 5% demonstrated a sensitivity of 0.54 (95% CI: 0.48–0.60, rank 10) while specificity increased to 0.66 (95% CI: 0.62–0.70, rank 6). PD-L1 ≥ 1% had the highest sensitivity among PD-L1 cutoffs (0.68, 95% CI: 0.65–0.71, rank 4) but the lowest specificity (0.48, 95% CI: 0.45–0.51, rank 12). TMB offered a balanced profile, with moderate sensitivity (0.56, 95% CI: 0.50–0.60, rank 9) but higher specificity (0.69, 95% CI: 0.65–0.73, rank 4). MSI status, with the highest specificity of 0.89 (95% CI: 0.85–0.93, rank 1), supports confirmatory testing in responders. However, it demonstrated low sensitivity at 0.36 (95% CI: 0.27–0.46, rank 13), indicating it may overlook a considerable number of actual responders. Among inflammatory markers, NLR showed sensitivity of 0.66 (95% CI: 0.57–0.74, rank 6) and specificity of 0.49 (95% CI: 0.42–0.56, rank 11). PLR exhibited sensitivity of 0.63 (95% CI: 0.51–0.73, rank 7) and specificity of 0.57 (95% CI: 0.47–0.66, rank 9). LIPI showed moderate sensitivity (0.57, 95% CI: 0.46–0.69, rank 8) and low specificity (0.54, 95% CI: 0.44–0.64, rank 10). LDH demonstrated sensitivity of 0.66 (95% CI: 0.55–0.76, rank 5) but the lowest specificity among all markers (0.40, 95% CI: 0.31–0.49, rank 13), making it the poorest overall performer. irAEs displayed relatively higher sensitivity (0.69, 95% CI: 0.60–0.77, rank 3) with moderate specificity (0.59, 95% CI: 0.50–0.67, rank 8), suggesting potential utility for treatment monitoring.

**Figure 3.**
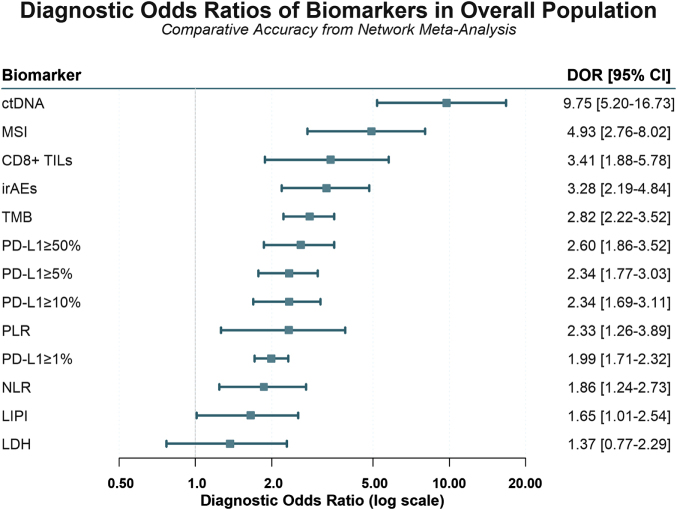
Forest plot of diagnostic odds ratios for biomarkers in the overall population. Each point represents the pooled diagnostic odds ratio from network meta-analysis for a biomarker, with horizontal lines indicating the 95% confidence interval. A DOR greater than 1 indicates diagnostic value, with higher values signifying better accuracy. ctDNA demonstrated the superior diagnostic performance (DOR = 9.75), while LDH showed no significant diagnostic value. DOR, diagnostic odds ratio; CI, confidence interval; ctDNA, circulating tumor DNA; CD8+ TILs, CD8-positive tumor-infiltrating lymphocytes; NLR, neutrophil-to-lymphocyte ratio; PD-L1, programmed death-ligand 1; LIPI, Lung Immune Prognostic Index; PLR, platelet-to-lymphocyte ratio; LDH, lactate dehydrogenase; TMB, tumor mutational burden; irAEs, immune-related adverse events; MSI, microsatellite instability.

In addition to sensitivity and specificity, multiple indicators (DOR, superiority, relative sensitivity, and relative specificity) were jointly evaluated to assess the ability of biomarkers to predict response to immunotherapy. The forest plot comparing the diagnostic odds ratios of the 13 biomarkers across the entire population is shown in Figure 3. According to the results of NMA, ctDNA also led with the highest DOR (9.75, 95% CI: 5.20–16.73, rank 1) and superiority (16.1, 95% CI: 9–21, rank 1), indicating robust discriminative power. MSI status, despite its low sensitivity, ranked second in DOR (4.93, 95% CI: 2.76–8.02, rank 2) and highest in relative specificity (1.86, 95% CI: 1.74–1.99, rank 1). CD8 + TILs ranked third in DOR (3.41, 95% CI: 1.88–5.78, rank 3) and second in superiority (3.26, 95% CI: 0.2–13, rank 2), reflecting moderate discriminatory power. irAEs also performed well, with a DOR of 3.28 (95% CI: 2.19–4.84, rank 4) and superiority of 3.01 (95% CI: 0.33–11, rank 3). TMB offered a DOR of 2.82 (95% CI: 2.22–3.52, rank 5) and superiority of 2.45 (95% CI: 0.33–7, rank 4), along with high relative specificity (1.44, 95% CI: 1.34–1.55, rank 4). PD-L1 diagnostic performance demonstrated a clear threshold dependency. PD-L1 ≥ 50% achieved a DOR of 2.60 (95% CI: 1.86–3.52, rank 6) and superiority of 1.42 (95% CI: 0.33–3, rank 6). At the ≥10% threshold, DOR was 2.34 (95% CI: 1.69–3.11, rank 7) with superiority of 0.95 (95% CI: 0.2–3, rank 8). Reducing the cutoff to ≥5% yielded a DOR of 2.34 (95% CI: 1.77–3.03, rank 8) and superiority of 0.90 (95% CI: 0.14–5, rank 9). Conversely, PD-L1 ≥ 1% demonstrated only modest diagnostic accuracy, with the lowest-ranked DOR (1.99, 95% CI: 1.71–2.32, rank 10) and superiority (0.57, 95% CI: 0.11–1.67, rank 10) among PD-L1 expression thresholds. Among inflammatory markers, PLR demonstrated a DOR of 2.33 (95% CI: 1.26–3.89, rank 9) and superiority of 1.11 (95% CI: 0.09–5, rank 7). In contrast, NLR and LIPI performed less robustly, with DOR values of 1.86 (95% CI: 1.24–2.73, rank 11) and 1.65 (95% CI: 1.01–2.54, rank 12), respectively. The poorest overall performer was LDH, with the lowest DOR (1.37, 95% CI: 0.77–2.29, rank 13) and superiority (0.18, 95% CI: 0.07–1, rank 13), highlighting its limited predictive value. These findings collectively emphasized ctDNA as the leading biomarker, while MSI, CD8 + TILs, PD-L1 ≥ 50%, and TMB served as valuable confirmatory tools in immunotherapy response prediction.

**Figure 4.**
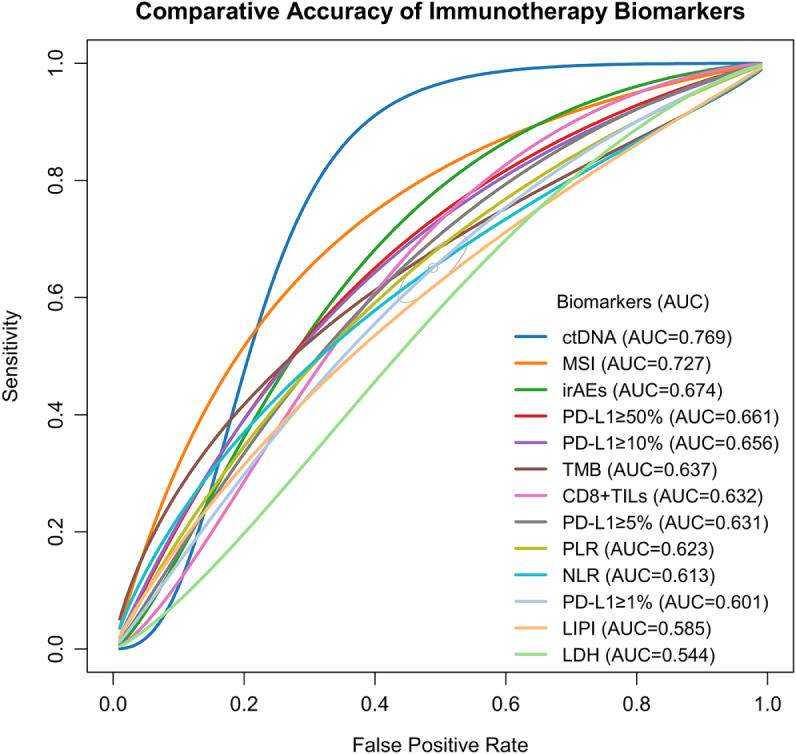
SROC curve for comparative accuracy of immunotherapy biomarkers. Comparison of prediction accuracy of immunotherapy biomarkers based on SROC curves and the AUC by meta-analysis. ctDNA, circulating tumor DNA; CD8+ TILs, CD8-positive tumor-infiltrating lymphocytes; NLR, neutrophil-to-lymphocyte ratio; PD-L1, programmed death-ligand 1; LIPI, Lung Immune Prognostic Index; PLR, platelet-to-lymphocyte ratio; LDH, lactate dehydrogenase; TMB, tumor mutational burden; irAEs, immune-related adverse events; MSI, microsatellite instability; SROC, summary receiver operating characteristic; AUC, area under the related curves.

Meanwhile, the AUC analysis revealed significant variation in predictive accuracy across biomarkers (Fig. 4). ctDNA demonstrated outstanding discriminative ability with the highest AUC of 0.769 as the top-performing marker. MSI followed with a strong AUC of 0.727. irAEs also showed relatively good performance with an AUC of 0.674. Among PD-L1 expression thresholds, predictive accuracy varied with cutoff levels: PD-L1 ≥ 50% achieved the highest AUC (0.661) among PD-L1 thresholds, followed by ≥10% (0.656) and ≥5% (0.631), while ≥1% performed the poorest (0.601). TMB and CD8+ TILs showed moderate predictive value, with AUC values of 0.637 and 0.632, respectively. Among inflammatory markers, PLR (AUC = 0.623) showed slightly better predictive power than NLR (0.613). In contrast, LIPI and LDH exhibited the least overall effectiveness, with AUC values of 0.585 and 0.544, respectively.

### Heterogeneity and publication bias

A key finding of the meta-analysis was the substantial heterogeneity observed across most studies. The pooled sensitivity (I^2^ = 82.5%) and specificity (I^2^ = 93.1%) both showed high inconsistency (*P* < 0.0001), which was especially pronounced for MSI, NLR, and LIPI (see Supplemental Digital Content Material 3, available at: http://links.lww.com/JS9/G519). Within the PD-L1 subgroup, significant heterogeneity was observed for both sensitivity (I^2^ range: 74.7%–88.8%) and specificity (I^2^ range: 82.9%–92.8%) across all expression cutoffs. In contrast, the overall DOR demonstrated low heterogeneity (I^2^ = 43.9%, *P* < 0.0001), indicating a more robust and consistent measure of discriminatory power. A fixed-effects model was employed for subgroups with three or fewer studies or low heterogeneity (I^2^ < 50%), revealing that the heterogeneity estimates remained consistent regardless of the model applied.

Deeks’ funnel plot asymmetry tests were performed to evaluate potential publication bias across all biomarker subgroups (Supplemental Digital Content Material 4, available at: http://links.lww.com/JS9/G519). Half of the biomarkers showed no significant evidence of publication bias at the 0.10 threshold, including CD8 + TILs (*P* = 0.328), ctDNA (*P* = 0.418), LDH (*P* = 0.444), PD-L1 ≥ 1% (*P* = 0.883), PD-L1 ≥ 5% (*P* = 0.141), PD-L1 ≥ 10% (*P* = 0.763), and TMB (*P* = 0.168). However, significant funnel plot asymmetry was detected for MSI (*P* = 0.082), irAEs (*P* = 0.005), LIPI (*P* = 0.095), NLR (*P* = 0.066), PLR (*P* = 0.074), and PD-L1 ≥ 50% (*P* = 0.02), suggesting the possibility of publication bias in the literature concerning these two biomarkers.

### Meta-regression analysis of heterogeneity sources for each biomarker

To investigate the potential sources of heterogeneity in the diagnostic performance of the biomarkers, univariable meta-regression analyses were performed. Several methodological and clinical covariates were tested, including treatment type, detection method, treatment line, cutoff derivation method, and scoring type. The results, visually summarized in Supplemental Digital Content Figure S2.1–S2.13, available at: http://links.lww.com/JS9/G519, revealed distinct patterns of heterogeneity influence across the different biomarkers.

Treatment type emerged as a predominant and strong source of heterogeneity, particularly for biomarkers related to the tumor immune microenvironment and treatment response. It accounted for a substantial proportion of the variance in sensitivity for CD8+ TILs (100%, *P* < 0.05), irAEs (52.27%, *P* < 0.01), LDH (47.99%, *P* < 0.05), and PD-L1 (50% positive cutoff: 33.16%, *P* < 0.05). This suggests that the class of immunotherapy or treatment regimen (monotherapy, dual immunotherapy, or combination therapy) significantly modulates the predictive power of these biomarkers. Similarly, the detection method was a major contributor, significantly explaining heterogeneity in sensitivity for MSI (51.77%, *P* < 0.01) and across various PD-L1 expression cutoffs (1%, 5%, and 10%), highlighting that the assay platform or technique used for biomarker measurement is a critical variable.

Other factors played more specific, context-dependent roles. The treatment line was a significant source of heterogeneity for the sensitivity of CD8+ TILs (64.44%, *P* < 0.05), indicating that the biomarker’s performance may differ between first-line and later-line settings. The method used to determine a biomarker’s positive cutoff was influential for the specificity of TMB (7.88%, *P* < 0.05) and showed a nonsignificant but considerable effect for PLR (29.60%, *P* = not significant). For PD-L1, the scoring type (e.g., tumor positive score vs. combined positive score) was a significant factor for specificity at the 1% cutoff (13.41%, *P* < 0.01). In contrast, for biomarkers such as ctDNA and NLR, none of the examined covariates significantly explained the observed heterogeneity, indicating that other unmeasured factors, such as cancer type or patient demographics, may be responsible.

In conclusion, the meta-regression analyses successfully identified several key drivers of heterogeneity. The most consistent factors were treatment type and detection method, underscoring the impact of therapeutic context and technical assay variation on biomarker performance. The influence of other factors, like treatment line and cutoff method, was more biomarker-specific. These findings emphasize the necessity of standardizing detection protocols and rigorously accounting for clinical covariates in future studies to ensure the reliable and comparable clinical application of these predictive biomarkers.

### Network meta-analysis for comparing biomarkers in subgroups

#### Subgroup analysis by tumor categories

**Figure 5.**
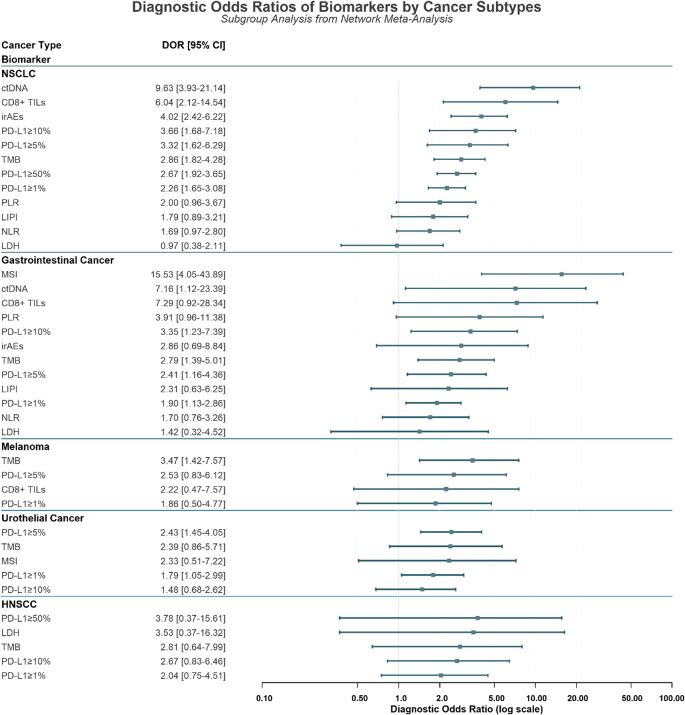
Forest plot of diagnostic odds ratios for biomarkers, stratified by cancer subtype. This forest plot presents the diagnostic odds ratios and 95% confidence intervals for various biomarkers, stratified by cancer type (NSCLC, Gastrointestinal Cancer, Melanoma, Urothelial Cancer, and HNSCC), derived from a network meta-analysis. Biomarkers are ranked within each cancer subtype from highest to lowest DOR. NSCLC, non-small lung cancer; HNSCC, head and neck squamous cell carcinoma; ctDNA: circulating tumor DNA; CD8+ TILs, CD8-positive tumor-infiltrating lymphocytes; NLR: neutrophil-to-lymphocyte ratio; PD-L1, programmed death-ligand 1; LIPI, Lung Immune Prognostic Index; PLR, platelet-to-lymphocyte ratio; LDH, lactate dehydrogenase; TMB, tumor mutational burden; irAEs, Immune-related adverse events; MSI, microsatellite instability; DOR, diagnostic odds ratio.

We conducted a network meta-analysis across five distinct cancer subtypes: NSCLC, melanoma, gastrointestinal (GI) cancer (a broad term for malignancies of the digestive tract, including esophageal, gastric, hepatocellular, pancreatic, and colorectal cancers), urothelial cancer, and head and neck squamous cell carcinoma (HNSCC). This selection represents the primary study populations in immunotherapy biomarker research, thereby ensuring statistically reliable subgroup analyses. The outcomes of the NMA analysis for each subgroup are presented in Table 2. The forest plot comparing the diagnostic odds ratios of the biomarkers across major cancer subtypes is presented in Figure 5.

**Table 2:** Subgroup network meta-analysis by cancer categories.

Markers	Number of studies	Sensitivity	RANK	Specificity	RANK	DOR	RANK	Superiority	RANK	Relative Sensitivity	RANK	Relative Specificity	RANK	AUC	RANK
(A) NSCLC															
ctDNA	7	0.77 (0.63, 0.88)	**1**	0.7 (0.57, 0.81)	**3**	9.63 (3.93, 21.14)	**1**	14.93 (3, 23)	**1**	1.05 (0.85, 1.22)	**1**	1.62 (1.27, 1.98)	**3**	0.754	**2**
irAEs	12	0.76 (0.66, 0.84)	**2**	0.55 (0.45, 0.65)	**7**	4.02 (2.42, 6.22)	**3**	4.15 (0.6, 11)	**3**	1.03 (0.88, 1.18)	**2**	1.26 (0.97, 1.58)	**7**	0.667	**5**
PD-L1 ≥ 1%	27	0.74 (0.68, 0.8)	**3**	0.43 (0.38, 0.49)	**11**	2.26 (1.65, 3.08)	**8**	0.95 (0.14, 3)	**7**	1 (1, 1)	**3**	1 (1, 1)	**11**	0.609	**9**
CD8 + TILs	3	0.72 (0.54, 0.86)	**4**	0.67 (0.51, 0.8)	**5**	6.04 (2.12, 14.54)	**2**	8.06 (0.33, 21)	**2**	0.96 (0.73, 1.17)	**4**	1.55 (1.18, 1.9)	**5**	0.782	**1**
LDH	3	0.64 (0.48, 0.8)	**5**	0.33 (0.2, 0.48)	**12**	0.97 (0.38, 2.11)	**12**	0.14 (0.05, 1)	**12**	0.86 (0.65, 1.07)	**5**	0.76 (0.46, 1.12)	**12**	0.409	**12**
NLR	9	0.62 (0.5, 0.74)	**6**	0.5 (0.39, 0.61)	**10**	1.69 (0.97, 2.8)	**11**	0.36 (0.07, 1.67)	**11**	0.83 (0.68, 0.99)	**6**	1.15 (0.89, 1.42)	**10**	0.577	**10**
PLR	6	0.62 (0.48, 0.76)	**7**	0.54 (0.41, 0.65)	**9**	2 (0.96, 3.67)	**9**	0.63 (0.07, 3)	**9**	0.83 (0.64, 1.02)	**8**	1.24 (0.94, 1.55)	**8**	0.705	**3**
PD-L1 ≥ 5%	5	0.62 (0.47, 0.74)	**8**	0.66 (0.54, 0.76)	**6**	3.32 (1.62, 6.29)	**5**	2.52 (0.11, 13)	**5**	0.83 (0.65, 1.01)	**7**	1.51 (1.22, 1.84)	**6**	0.623	**8**
LIPI	5	0.59 (0.44, 0.74)	**9**	0.54 (0.4, 0.66)	**8**	1.79 (0.89, 3.21)	**10**	0.42 (0.06, 1.8)	**10**	0.8 (0.59, 1)	**9**	1.23 (0.9, 1.6)	**9**	0.572	**11**
TMB	14	0.56 (0.46, 0.65)	**10**	0.68 (0.6, 0.75)	**4**	2.86 (1.82, 4.28)	**6**	1.43 (0.11, 7)	**6**	0.76 (0.62, 0.89)	**10**	1.57 (1.33, 1.83)	**4**	0.656	**7**
PD-L1 ≥ 10%	5	0.54 (0.39, 0.7)	**11**	0.74 (0.63, 0.83)	**2**	3.66 (1.68, 7.18)	**4**	3.22 (0.14, 13)	**4**	0.72 (0.53, 0.92)	**11**	1.71 (1.42, 2.01)	**2**	0.697	**4**
PD-L1 ≥ 50%	21	0.47 (0.4, 0.54)	**12**	0.75 (0.7, 0.8)	**1**	2.67 (1.92, 3.65)	**7**	0.93 (0.14, 3)	**8**	0.63 (0.54, 0.71)	**12**	1.73 (1.53, 1.96)	**1**	0.659	**6**
(B) Gastrointestinal cancer															
ctDNA	3	0.77 (0.51, 0.94)	**1**	0.58 (0.34, 0.8)	**9**	7.16 (1.12, 23.39)	**3**	6.4 (0.14, 19)	**1**	1.39 (0.91, 1.83)	**1**	0.98 (0.58, 1.37)	**9**	0.704	**2**
CD8+ TILs	2	0.75 (0.44, 0.95)	**2**	0.57 (0.3, 0.82)	**10**	7.29 (0.92, 28.34)	**2**	5.79 (0.09, 17)	**2**	1.36 (0.78, 1.85)	**2**	0.97 (0.52, 1.41)	**10**	0.676	**4**
LDH	3	0.66 (0.42, 0.87)	**3**	0.36 (0.18, 0.55)	**12**	1.42 (0.32, 4.52)	**12**	0.48 (0.06, 3)	**12**	1.2 (0.73, 1.69)	**3**	0.61 (0.31, 0.98)	**12**	0.364	**12**
NLR	8	0.64 (0.49, 0.78)	**4**	0.47 (0.33, 0.61)	**11**	1.7 (0.76, 3.26)	**11**	0.61 (0.08, 3)	**11**	1.16 (0.84, 1.52)	**4**	0.79 (0.54, 1.07)	**11**	0.596	**7**
PLR	6	0.63 (0.39, 0.83)	**5**	0.64 (0.44, 0.81)	**6**	3.91 (0.96, 11.38)	**4**	4.69 (0.09, 15)	**3**	1.14 (0.69, 1.6)	**5**	1.09 (0.72, 1.43)	**6**	0.686	**3**
PD-L1 ≥ 1%	26	0.56 (0.46, 0.65)	**6**	0.59 (0.51, 0.67)	**8**	1.9 (1.13, 2.86)	**10**	0.82 (0.09, 5)	**10**	1 (1, 1)	**6**	1 (1, 1)	**8**	0.601	**6**
TMB	20	0.55 (0.42, 0.68)	**7**	0.68 (0.58, 0.78)	**5**	2.79 (1.39, 5.01)	**7**	2.88 (0.11, 11)	**4**	0.99 (0.73, 1.29)	**7**	1.16 (0.96, 1.38)	**5**	0.564	**10**
LIPI	3	0.53 (0.3, 0.77)	**8**	0.63 (0.42, 0.8)	**7**	2.31 (0.63, 6.25)	**9**	1.86 (0.07, 11)	**8**	0.96 (0.53, 1.45)	**8**	1.06 (0.69, 1.44)	**7**	0.577	**8**
irAEs	3	0.49 (0.25, 0.77)	**9**	0.69 (0.49, 0.85)	**4**	2.86 (0.69, 8.84)	**6**	2.77 (0.08, 13)	**5**	0.89 (0.43, 1.46)	**9**	1.18 (0.82, 1.53)	**4**	0.473	**11**
PD-L1 ≥ 5%	11	0.46 (0.34, 0.59)	**10**	0.73 (0.61, 0.81)	**3**	2.41 (1.16, 4.36)	**8**	1.7 (0.11, 9)	**9**	0.83 (0.59, 1.1)	**10**	1.23 (0.97, 1.47)	**3**	0.632	**5**
PD-L1 ≥ 10%	6	0.37 (0.22, 0.54)	**11**	0.84 (0.73, 0.91)	**2**	3.35 (1.23, 7.39)	**5**	1.89 (0.2, 9)	**7**	0.66 (0.4, 1.01)	**11**	1.41 (1.18, 1.65)	**2**	0.809	**1**
MSI	5	0.3 (0.16, 0.5)	**12**	0.96 (0.93, 0.99)	**1**	15.53 (4.05, 43.89)	**1**	2.2 (1, 9)	**6**	0.55 (0.29, 0.91)	**12**	1.63 (1.42, 1.89)	**1**	0.575	**9**
(C) Melanoma															
PD-L1 ≥ 1%	5	0.54 (0.3, 0.75)	**1**	0.57 (0.4, 0.73)	**4**	1.86 (0.5, 4.77)	**4**	0.95 (0.2, 5)	**4**	1 (1, 1)	**1**	1 (1, 1)	**4**	0.557	**4**
TMB	10	0.52 (0.35, 0.7)	**2**	0.74 (0.62, 0.84)	**1**	3.47 (1.42, 7.57)	**1**	3.51 (1, 7)	**1**	1 (0.63, 1.71)	**2**	1.33 (1.02, 1.84)	**1**	0.661	**1**
CD8 + TILs	4	0.49 (0.24, 0.78)	**3**	0.64 (0.42, 0.8)	**3**	2.22 (0.47, 7.57)	**3**	1.14 (0.2, 5)	**3**	0.94 (0.45, 1.76)	**3**	1.14 (0.73, 1.63)	**3**	0.562	**3**
PD-L1 ≥ 5%	9	0.48 (0.29, 0.67)	**4**	0.71 (0.54, 0.83)	**2**	2.53 (0.83, 6.12)	**2**	1.85 (0.33, 7)	**2**	0.91 (0.5, 1.6)	**4**	1.27 (0.91, 1.75)	**2**	0.625	**2**
(D) Urothelial cancer															
PD-L1 ≥ 1%	10	0.76 (0.64, 0.86)	**1**	0.35 (0.24, 0.47)	**5**	1.79 (1.05, 2.99)	**4**	0.98 (0.33, 1)	**4**	1 (1, 1)	**1**	1 (1, 1)	**5**	0.585	**3**
TMB	3	0.64 (0.42, 0.81)	**2**	0.53 (0.35, 0.73)	**4**	2.39 (0.86, 5.71)	**2**	1.43 (0.33, 5)	**3**	0.84 (0.6, 1.04)	**2**	1.56 (1.06, 2.21)	**4**	0.536	**4**
PD-L1 ≥ 5%	9	0.59 (0.45, 0.72)	**3**	0.61 (0.49, 0.72)	**3**	2.43 (1.45, 4.05)	**1**	1.81 (0.33, 5)	**1**	0.78 (0.64, 0.89)	**3**	1.8 (1.4, 2.29)	**3**	0.676	**1**
PD-L1 ≥ 10%	7	0.41 (0.25, 0.6)	**4**	0.66 (0.51, 0.79)	**2**	1.48 (0.68, 2.62)	**5**	0.9 (0.2, 3)	**5**	0.54 (0.35, 0.79)	**4**	1.97 (1.3, 2.72)	**2**	0.609	**2**
MSI	2	0.34 (0.12, 0.61)	**5**	0.78 (0.57, 0.92)	**1**	2.33 (0.51, 7.22)	**3**	1.45 (0.33, 3)	**2**	0.45 (0.16, 0.78)	**5**	2.29 (1.58, 3.23)	**1**	0.185	**5**
(E) HNSCC															
LDH	2	0.74 (0.44, 0.94)	**1**	0.41 (0.17, 0.7)	**4**	3.53 (0.37, 16.32)	**2**	1.6 (0.14, 5)	**2**	1.04 (0.6, 1.44)	**1**	1.03 (0.39, 1.92)	**4**	0.684	**2**
PD-L1 ≥ 1%	11	0.72 (0.58, 0.85)	**2**	0.41 (0.27, 0.55)	**5**	2.04 (0.75, 4.51)	**5**	1.2 (0.2, 3)	**5**	1 (1, 1)	**2**	1 (1, 1)	**5**	0.575	**5**
PD-L1 ≥ 10%	7	0.55 (0.36, 0.73)	**3**	0.65 (0.48, 0.8)	**3**	2.67 (0.83, 6.46)	**4**	1.58 (0.2, 5)	**3**	0.76 (0.49, 1.1)	**3**	1.64 (1.04, 2.49)	**3**	0.648	**4**
PD-L1 ≥ 50%	2	0.46 (0.18, 0.77)	**4**	0.73 (0.46, 0.91)	**2**	3.78 (0.37, 15.61)	**1**	1.96 (0.2, 7)	**1**	0.63 (0.24, 1.11)	**4**	1.85 (1.01, 2.81)	**2**	0.68	**3**
TMB	5	0.4 (0.2, 0.63)	**5**	0.77 (0.58, 0.89)	**1**	2.81 (0.64, 7.99)	**3**	1.54 (0.2, 5)	**4**	0.56 (0.28, 0.91)	**5**	1.94 (1.28, 2.86)	**1**	0.73	**1**

Based on the network meta-analysis (NMA), the diagnostic efficacy (sensitivity, specificity, DOR, and other indicators and ranking) of immunotherapy biomarkers in four cancer subtypes (NSCLC, gastrointestinal cancer, melanoma, urothelial cancer, and HNSCC) was compared. AUC, area under curve; CD8+ TILs, CD8-positive tumor-infiltrating lymphocytes; ctDNA, circulating tumor DNA; DOR, diagnostic odds ratio; HNSCC, head and neck squamous cell carcinoma; irAEs, immune-related adverse events; LDH, lactate dehydrogenase; LIPI, Lung Immune Prognostic Index; MSI, microsatellite instability; NLR, neutrophil-to-lymphocyte ratio; NSCLC, non-small lung cancer; PD-L1, programmed death-ligand 1; PLR, platelet-to-lymphocyte ratio; TMB, tumor mutational burden.

For NSCLC, ctDNA emerged as the most sensitive biomarker (0.77, 95% CI: 0.63–0.88, rank 1), followed by irAEs at 0.76 (95% CI: 0.66–0.84, rank 2). Regarding specificity, PD-L1 ≥ 50% ranked highest at 0.75 (95% CI: 0.70–0.80, rank 1), while PD-L1 ≥ 10% followed closely at 0.74 (95% CI: 0.63–0.83, rank 2). In terms of diagnostic accuracy, ctDNA dominated with the highest DOR (9.63, 95% CI: 3.93–21.14, rank 1) and ranked second in AUC (0.754, rank 2). CD8+ TILs achieved the highest AUC (0.782, rank 1) and ranked second in DOR (6.04, 95% CI: 2.12–14.54). PD-L1 ≥ 10% also performed well, ranking fourth in DOR (3.66, 95% CI: 1.68–7.18) and fourth in AUC (0.697, rank 4). Notably, PD-L1 ≥ 5% showed moderate specificity (0.66, 95% CI: 0.54–0.76, rank 6) and a competitive DOR (3.32, 95% CI: 1.62–6.29, rank 5), with an AUC of 0.623 (rank 8).

For gastrointestinal cancer, ctDNA exhibited the highest sensitivity (0.77, 95% CI: 0.51–0.94, rank 1), followed by CD8+ TILs (0.75, 95% CI: 0.44–0.95, rank 2). MSI status ranked highest in specificity (0.96, 95% CI: 0.93–0.99, rank 1) and DOR (15.53, 95% CI: 4.05–43.89, rank 1), while PD-L1 ≥ 10% showed the best AUC (0.809, rank 1). ctDNA also performed well in DOR (7.16, 95% CI: 1.12–23.39, rank 3) and ranked second in AUC (0.704, rank 2), highlighting its potential for monitoring treatment response.

In melanoma, PD-L1 ≥ 1% demonstrated the highest sensitivity (0.54, 95% CI: 0.30–0.75, rank 1), followed by TMB (0.52, 95% CI: 0.35–0.70, rank 2). For specificity, TMB ranked first (0.74, 95% CI: 0.62–0.84, rank 1), followed by PD-L1 ≥ 5% (0.71, 95% CI: 0.54–0.83, rank 2). TMB also led in DOR (3.47, 95% CI: 1.42–7.57, rank 1) and achieved the best AUC (0.661, rank 1). PD-L1 ≥ 5% showed balanced performance with a DOR of 2.53 (95% CI: 0.83–6.12, rank 2) and an AUC of 0.625 (rank 2), reflecting its diagnostic relevance in this subgroup.

In urothelial cancer, PD-L1 ≥ 1% ranked first in sensitivity (0.76, 95% CI: 0.64–0.86, rank 1), while MSI achieved the highest specificity (0.78, 95% CI: 0.57–0.92, rank 1). For diagnostic accuracy, PD-L1 ≥ 5% led to DOR (2.43, 95% CI: 1.45–4.05, rank 1) and AUC (0.676, rank 1). PD-L1 ≥ 10% also performed well, ranking second in AUC (0.609, rank 2) and specificity (0.66, 95% CI: 0.51–0.79, rank 2). TMB showed moderate performance with a sensitivity of 0.64 (95% CI: 0.42–0.81, rank 2) and an AUC of 0.536 (rank 4).

In HNSCC, LDH demonstrated the highest sensitivity (0.74, 95% CI: 0.44–0.94, rank 1), followed by PD-L1 ≥ 1% (0.72, 95% CI: 0.58–0.85, rank 2). For specificity, TMB ranked first (0.77, 95% CI: 0.58–0.89, rank 1), with PD-L1 ≥ 50% following closely (0.73, 95% CI: 0.46–0.91, rank 2). TMB also achieved the highest AUC (0.73, rank 1), while PD-L1 ≥ 50% led in DOR (3.78, 95% CI: 0.37–15.61, rank 1). LDH showed balanced diagnostic performance, ranking second in both DOR (3.53, 95% CI: 0.37–16.32) and AUC (0.684), underscoring its potential utility in this cancer type.

**Figure 6.**
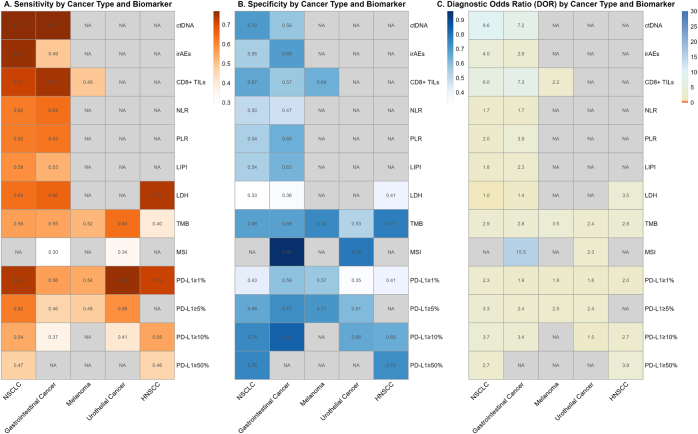
Performance of immunotherapy predictive biomarkers stratified by cancer type. This heat map illustrates the cancer type-specific performance of major immunotherapy predictive biomarkers. (A) Sensitivity, (B) specificity, and (C) diagnostic odds ratio (DOR) are displayed for each biomarker across different solid tumors, including non-small cell lung cancer (NSCLC), gastrointestinal cancer, melanoma, urothelial cancer, and head and neck squamous cell carcinoma (HNSCC). Color intensity corresponds to the performance metric value, with darker shades generally indicating higher values for each parameter. Blank cells (NA) indicate insufficient data for that specific biomarker-cancer combination.

Across the cancer subtypes evaluated, a heatmap (Fig. 6) illustrating biomarker sensitivity and specificity by cancer type reveals distinct patterns of diagnostic utility. ctDNA demonstrates consistently strong performance in NSCLC, leading in multiple key metrics, while also showing high sensitivity in gastrointestinal cancer. TMB maintains robust specificity in both melanoma and urothelial cancer, and leads in diagnostic accuracy in melanoma. PD-L1 expression thresholds showed variable, yet context-dependent clinical utility in other cancer types. MSI status stood out as a highly specific biomarker in gastrointestinal cancer, while irAEs displayed higher specificity in gastrointestinal cancer compared to NSCLC. These patterns highlight the importance of context-specific biomarker selection based on cancer type and clinical objectives. Heterogeneity differed significantly across subgroups (detailed in Supplemental Digital Content Material 5.1–5.5, available at: http://links.lww.com/JS9/G519), with pronounced variability observed in NSCLC (I^2^ = 86.3%) and moderate consistency in GI cancer (I^2^ = 62.6%) for sensitivity. Collectively, these results underscore the critical importance of biomarker-specific and cancer-type-specific validation for predicting immunotherapy response.

#### Subgroup analysis by immunotherapy regimen settings

To comprehensively assess the diagnostic performance of biomarkers under different therapeutic strategies, we performed a subgroup network meta-analysis stratified into four distinct immunotherapy settings: immunotherapy monotherapy, chemo-immunotherapy combination, dual immunotherapy combination, and immunotherapy-targeted therapy combination. The outcomes of this analysis are presented in Table 3.

**Table 3:** Subgroup network meta-analysis by immunotherapy settings.

Markers	Number of studies	Sensitivity	RANK	Specificity	RANK	DOR	RANK	Superiority	RANK	Relative Sensitivity	RANK	Relative Specificity	RANK	AUC	RANK	
(A) Immunotherapy monotherapy																
ctDNA	4	0.79 (0.62, 0.91)	**1**	0.65 (0.47, 0.8)	**7**	9.01 (2.83, 23)	**1**	12.09 (1, 23)	**1**	1.09 (0.86, 1.27)	**1**	1.38 (0.99, 1.73)	**7**	0.809	**1**	
LDH	7	0.74 (0.61, 0.84)	**2**	0.4 (0.29, 0.51)	**13**	2.01 (0.92, 3.83)	**12**	0.51 (0.08, 3)	**13**	1.01 (0.83, 1.17)	**2**	0.85 (0.61, 1.11)	**13**	0.618	**12**	
PD-L1 ≥ 1%	67	0.73 (0.69, 0.77)	**3**	0.47 (0.43, 0.51)	**12**	2.41 (1.91, 2.97)	**10**	0.8 (0.11, 3)	**11**	1 (1, 1)	**3**	1 (1, 1)	**12**	0.623	**9**	
CD8 + TILs	11	0.73 (0.61, 0.83)	**4**	0.59 (0.48, 0.7)	**9**	4.15 (2.02, 7.88)	**4**	4.21 (0.2, 13)	**3**	1 (0.82, 1.15)	**4**	1.25 (1.01, 1.49)	**9**	0.718	**3**	
NLR	10	0.71 (0.6, 0.8)	**5**	0.49 (0.39, 0.6)	**11**	2.47 (1.33, 4.28)	**8**	0.98 (0.09, 5)	**9**	0.97 (0.82, 1.11)	**5**	1.05 (0.82, 1.3)	**11**	0.639	**6**	
irAEs	10	0.67 (0.54, 0.79)	**6**	0.67 (0.56, 0.77)	**5**	4.44 (2.4, 7.66)	**3**	5.75 (0.33, 15)	**2**	0.92 (0.74, 1.1)	**6**	1.43 (1.17, 1.68)	**5**	0.699	**4**	
PLR	6	0.63 (0.44, 0.78)	**7**	0.55 (0.39, 0.71)	**10**	2.34 (0.88, 5.36)	**11**	1.04 (0.06, 9)	**8**	0.86 (0.61, 1.09)	**7**	1.17 (0.82, 1.51)	**10**	0.619	**11**	
PD-L1 ≥ 5%	31	0.56 (0.49, 0.63)	**8**	0.66 (0.6, 0.71)	**6**	2.46 (1.71, 3.43)	**9**	0.81 (0.11, 5)	**10**	0.77 (0.66, 0.87)	**8**	1.39 (1.24, 1.55)	**6**	0.653	**8**	
LIPI	4	0.54 (0.36, 0.73)	**9**	0.59 (0.42, 0.76)	**8**	1.92 (0.77, 4.1)	**13**	0.62 (0.06, 5)	**12**	0.75 (0.49, 1.01)	**9**	1.26 (0.88, 1.63)	**8**	0.618	**12**	
TMB	40	0.54 (0.47, 0.61)	**10**	0.71 (0.66, 0.76)	**4**	2.9 (2.06, 3.97)	**6**	2.18 (0.14, 7)	**5**	0.74 (0.64, 0.85)	**10**	1.51 (1.36, 1.68)	**4**	0.626	**10**	
PD-L1 ≥ 50%	12	0.51 (0.4, 0.62)	**11**	0.74 (0.66, 0.81)	**2**	3.17 (1.8, 5.15)	**5**	3.04 (0.14, 11)	**4**	0.7 (0.55, 0.86)	**11**	1.58 (1.39, 1.77)	**2**	0.692	**5**	
PD-L1 ≥ 10%	26	0.48 (0.4, 0.56)	**12**	0.73 (0.67, 0.78)	**3**	2.52 (1.71, 3.5)	**7**	1.16 (0.11, 5)	**7**	0.66 (0.56, 0.77)	**12**	1.55 (1.39, 1.7)	**3**	0.658	**7**	
MSI	12	0.36 (0.26, 0.47)	**13**	0.9 (0.85, 0.94)	**1**	5.25 (2.81, 9.4)	**2**	1.23 (1, 3)	**6**	0.49 (0.36, 0.65)	**13**	1.91 (1.74, 2.08)	**1**	0.723	**2**	
(B) Chemo-immunotherapy combination																
ctDNA	3	0.8 (0.64, 0.92)	**1**	0.68 (0.44, 0.87)	**2**	11.26 (2.95, 30.42)	**1**	10.15 (1.67, 15)	**1**	1.27 (1, 1.55)	**1**	1.65 (1.06, 2.25)	**2**	0.771	**2**	
PLR	2	0.7 (0.45, 0.89)	**2**	0.59 (0.39, 0.76)	**5**	4.65 (1.16, 13.45)	**2**	4.41 (0.33, 11)	**2**	1.11 (0.72, 1.46)	**2**	1.44 (0.96, 1.93)	**5**	0.794	**1**	
PD-L1 ≥ 1%	14	0.63 (0.53, 0.73)	**3**	0.41 (0.33, 0.51)	**8**	1.26 (0.82, 1.89)	**7**	0.33 (0.14, 1)	**7**	1 (1, 1)	**3**	1 (1, 1)	**8**	0.517	**7**	
TMB	3	0.61 (0.44, 0.76)	**4**	0.57 (0.4, 0.73)	**6**	2.28 (0.89, 5.15)	**3**	1.83 (0.14, 9)	**4**	0.96 (0.72, 1.19)	**4**	1.38 (0.99, 1.84)	**6**	0.528	**6**	
NLR	3	0.51 (0.3, 0.71)	**5**	0.59 (0.38, 0.77)	**4**	1.77 (0.55, 4.33)	**5**	1.51 (0.11, 7)	**5**	0.81 (0.51, 1.09)	**5**	1.44 (0.94, 1.91)	**4**	0.597	**4**	
PD-L1 ≥ 5%	6	0.41 (0.27, 0.56)	**6**	0.65 (0.53, 0.77)	**3**	1.37 (0.69, 2.35)	**6**	1.18 (0.11, 5)	**6**	0.64 (0.43, 0.88)	**6**	1.59 (1.2, 2.01)	**3**	0.587	**5**	
PD-L1 ≥ 50%	5	0.35 (0.24, 0.47)	**7**	0.76 (0.65, 0.85)	**1**	1.84 (1, 3.19)	**4**	2.2 (0.33, 5)	**3**	0.55 (0.43, 0.69)	**7**	1.86 (1.52, 2.25)	**1**	0.708	**3**	
LDH	2	0.33 (0.15, 0.53)	**8**	0.49 (0.29, 0.68)	**7**	0.53 (0.16, 1.27)	**8**	0.12 (0.07, 0.33)	**8**	0.52 (0.26, 0.8)	**8**	1.2 (0.74, 1.67)	**7**	0.402	**8**	
(C) Dual immunotherapy combination																
TMB	6	0.67 (0.54, 0.8)	**1**	0.61 (0.5, 0.72)	**3**	3.52 (1.64, 7.08)	**1**	3.7 (0.33, 7)	**1**	1.16 (0.9,1.5)	**1**	1.1 (0.9, 1.33)	**3**	0.603	**4**	
LIPI	2	0.64 (0.41, 0.84)	**2**	0.36 (0.2, 0.55)	**5**	1.19 (0.36, 3.06)	**5**	0.58 (0.14, 1)	**5**	1.11 (0.67, 1.59)	**2**	0.65 (0.36, 1.01)	**5**	0.509	**5**	
PD-L1 ≥ 1%	12	0.58 (0.46, 0.69)	**3**	0.56 (0.47, 0.65)	**4**	1.87 (1.04, 3.06)	**4**	0.83 (0.2, 3)	**4**	1 (1, 1)	**3**	1 (1, 1)	**4**	0.619	**2**	
PD-L1 ≥ 5%	2	0.55 (0.3, 0.75)	**4**	0.67 (0.49, 0.82)	**2**	3.02 (0.88, 7.92)	**2**	2.15 (0.2, 7)	**2**	0.94 (0.5, 1.39)	**4**	1.21 (0.86, 1.55)	**2**	0.616	**3**	
PD-L1 ≥ 50%	3	0.32 (0.19, 0.49)	**5**	0.84 (0.75, 0.9)	**1**	2.76 (1.15, 5.75)	**3**	1.17 (0.98, 3)	**3**	0.55 (0.35, 0.83)	**5**	1.51 (1.29, 1.75)	**1**	0.644	**1**	
(D) Immunotherapy-targeted therapy combination																
PD-L1 ≥ 5%	4	0.53 (0.31, 0.74)	**1**	0.76 (0.59, 0.9)	**1**	4.68 (1.22, 13.09)	**1**	0.53 (0.31, 0.74)	**1**	1.09 (0.64, 1.68)	**1**	1.32 (0.93, 1.78)	**1**	0.699	**1**	
PD-L1 ≥ 1%	8	0.5 (0.34, 0.65)	**2**	0.59 (0.44, 0.72)	**4**	1.58 (0.63, 3.39)	**4**	0.5 (0.34, 0.65)	**3**	1 (1, 1)	**2**	1 (1, 1)	**4**	0.575	**3**	
TMB	7	0.46 (0.3, 0.62)	**3**	0.71 (0.57, 0.83)	**2**	2.37 (0.88, 5.06)	**2**	0.46 (0.3, 0.62)	**2**	0.93 (0.61, 1.38)	**3**	1.22 (0.92, 1.58)	**3**	0.608	**2**	
PD-L1 ≥ 10%	3	0.34 (0.16, 0.57)	**4**	0.71 (0.49, 0.88)	**3**	1.62 (0.35, 5.25)	**3**	0.34 (0.16, 0.57)	**4**	0.69 (0.32, 1.24)	**4**	1.22 (0.81, 1.69)	**2**	0.567	**4**	

Based on the network meta-analysis (NMA), the diagnostic efficacy (sensitivity, specificity, DOR, and other indicators and ranking) of immunotherapy biomarkers in four different immunotherapy settings (immunotherapy monotherapy; chemo-immunotherapy combination; dual immunotherapy combination; immunotherapy-targeted therapy combination) was compared. AUC, area under curve; CD8+ TILs, CD8-positive tumor-infiltrating lymphocytes; ctDNA, circulating tumor DNA; DOR, diagnostic odds ratio; irAEs, immune-related adverse events; LDH, lactate dehydrogenase; LIPI, Lung Immune Prognostic Index; MSI, microsatellite instability; NLR, neutrophil-to-lymphocyte ratio; PD-L1, programmed death-ligand 1; PLR, platelet-to-lymphocyte ratio; TMB, tumor mutational burden.

In the monotherapy setting, ctDNA emerged as the top-performing biomarker, ranking first in sensitivity (0.79, 95% CI: 0.62–0.91), DOR (9.01, 95% CI: 2.83–23.00), superiority (12.09, 95% CI: 1–23), and AUC (0.809). However, its specificity was moderate (0.65, 95% CI: 0.47–0.80). In contrast, MSI demonstrated the highest specificity (0.90, 95% CI: 0.85–0.94) and relative specificity (1.91, 95% CI: 1.74–2.08), ranking first in both, but it had the lowest sensitivity (0.36, 95% CI: 0.26–0.47). Biomarkers such as CD8 + TILs showed a balanced profile with good sensitivity (0.73, 95% CI: 0.61–0.83) and DOR (4.15, 95% CI: 2.02–7.88), while irAEs offered higher specificity (0.67, 95% CI: 0.56–0.77) and was the second most superior marker (5.75, 95% CI: 0.33–15). The performance of PD-L1 expression varied with cutoff levels; higher cutoffs (≥50%) also predicted better specificity but poorer sensitivity.

Within the chemo-immunotherapy subgroup, ctDNA again consistently ranked first in sensitivity (0.80, 95% CI: 0.64–0.92), DOR (11.26, 95% CI: 2.95–30.42), and superiority (10.15, 95% CI: 1.67–15). Notably, PLR showed a remarkable performance, achieving the highest AUC (0.794) and ranking second in DOR (4.65, 95% CI: 1.16–13.45) and superiority (4.41, 95% CI: 0.33–11). PD-L1 ≥ 50% provided the highest specificity (0.76, 95% CI: 0.65–0.85) and relative specificity (1.86, 95% CI: 1.52–2.25), albeit with low sensitivity (0.35, 95% CI: 0.24–0.47). LDH performed poorly across most metrics, showing the lowest DOR (0.53, 95% CI: 0.16–1.27) and superiority (0.12, 95% CI: 0.07–0.33) in this setting.

For dual immunotherapy regimens, TMB was the leading biomarker, ranking first in sensitivity (0.67, 95% CI: 0.54–0.80), DOR (3.52, 95% CI: 1.64–7.08), and superiority (3.70, 95% CI: 0.33–7). PD-L1 ≥ 50% exhibited the highest specificity (0.84, 95% CI: 0.75–0.90) and the highest AUC (0.644) in this subgroup, despite having the lowest sensitivity (0.32, 95% CI: 0.19–0.49). PD-L1 ≥ 5% also showed a promising DOR (3.02, 95% CI: 0.88–7.92) and ranked second in superiority (2.15, 95% CI: 0.20–7). LIPI, while ranking second in sensitivity (0.64, 95% CI: 0.41–0.84), had low specificity (0.36, 95% CI: 0.20–0.55) and a low DOR (1.19, 95% CI: 0.36–3.06).

In the immunotherapy-targeted therapy combination setting, PD-L1 ≥ 5% demonstrated the most robust overall performance, ranking first in sensitivity (0.53, 95% CI: 0.31–0.74), specificity (0.76, 95% CI: 0.59–0.90), DOR (4.68, 95% CI: 1.22–13.09), and AUC (0.699). TMB presented with the second-highest specificity (0.71, 95% CI: 0.57–0.83) and AUC (0.608), though its sensitivity was moderate (0.46, 95% CI: 0.30–0.62). PD-L1 ≥ 10% had the lowest sensitivity (0.34, 95% CI: 0.16–0.57) but maintained a relatively high specificity (0.71, 95% CI: 0.49–0.88).

This analysis highlights that the effectiveness of immunotherapy biomarkers varies based on the treatment regimen. ctDNA showed superior sensitivity and diagnostic power in both immunotherapy monotherapy and chemo-immunotherapy combination settings. MSI and high PD-L1 cutoffs (≥50%) were the best for specificity overall. PLR excelled in the chemo-immunotherapy combination subgroup, achieving the highest AUC, while TMB was the top biomarker in dual immunotherapy settings. LDH generally underperformed across metrics. In summary, no single biomarker is universally best. The optimal choice depends on the treatment strategy, emphasizing the need for context-specific biomarker selection in clinical practice and trials.

#### Subgroup analysis by treatment lines

To evaluate the influence of treatment lines on the predictive value of biomarkers, we conducted a subgroup network meta-analysis stratified into two cohorts: first-line treatment and second-line or later-line treatment. The results of this subgroup analysis are summarized in Table 4.

**Table 4:** Subgroup network meta-analysis by treatment lines.

Markers	Number of studies	Sensitivity	RANK	Specificity	RANK	DOR	RANK	Superiority	RANK	Relative Sensitivity	RANK	Relative Specificity	RANK	AUC	RANK		
(A) First-line																	
irAEs	4	0.9 (0.83, 0.95)	**1**	0.25 (0.16, 0.38)	**10**	3.34 (1.82, 5.89)	**2**	1.2 (0.33, 3)	**6**	1.37 (1.23, 1.51)	**1**	0.58 (0.37, 0.86)	**10**	0.657	**2**		
ctDNA	3	0.8 (0.65, 0.9)	**2**	0.59 (0.39, 0.79)	**6**	6.73 (2.54, 15.03)	**1**	7.96 (1, 15)	**1**	1.21 (0.98, 1.4)	**2**	1.35 (0.89, 1.89)	**6**	0.771	**1**		
PD-L1 ≥ 1%	31	0.66 (0.6, 0.72)	**3**	0.44 (0.38, 0.5)	**8**	1.54 (1.25, 1.87)	**8**	0.57 (0.2, 3)	**8**	1 (1, 1)	**3**	1 (1, 1)	**8**	0.585	**8**		
LIPI	3	0.63 (0.37, 0.85)	**4**	0.41 (0.19, 0.64)	**9**	1.23 (0.68, 2.01)	**9**	0.38 (0.11, 1)	**9**	0.95 (0.55, 1.31)	**4**	0.93 (0.43, 1.51)	**9**	0.552	**9**		
TMB	24	0.6 (0.52, 0.67)	**5**	0.6 (0.53, 0.67)	**5**	2.24 (1.68, 2.93)	**3**	2.27 (0.33, 7)	**3**	0.91 (0.82, 1)	**5**	1.36 (1.22, 1.53)	**5**	0.643	**3**		
CD8 + TILs	4	0.55 (0.41, 0.69)	**6**	0.6 (0.47, 0.73)	**4**	2 (0.96, 3.69)	**5**	1.99 (0.14, 7)	**5**	0.83 (0.63, 1.05)	**6**	1.38 (1.07, 1.71)	**4**	0.603	**7**		
PD-L1 ≥ 5%	19	0.48 (0.39, 0.56)	**7**	0.67 (0.6, 0.74)	**3**	1.87 (1.38, 2.44)	**6**	2.18 (0.33, 5)	**4**	0.72 (0.6, 0.83)	**7**	1.53 (1.35, 1.74)	**3**	0.612	**6**		
NLR	4	0.42 (0.3, 0.55)	**8**	0.58 (0.46, 0.7)	**7**	1.06 (0.62, 1.71)	**10**	0.25 (0.09, 1)	**10**	0.64 (0.47, 0.82)	**8**	1.33 (1.06, 1.61)	**7**	0.542	**10**		
PD-L1 ≥ 50%	10	0.39 (0.31, 0.46)	**9**	0.77 (0.72, 0.82)	**1**	2.17 (1.61, 2.91)	**4**	2.42 (0.33, 5)	**2**	0.58 (0.5, 0.67)	**9**	1.77 (1.6, 1.97)	**1**	0.632	**4**		
PD-L1 ≥ 10%	11	0.34 (0.26, 0.44)	**10**	0.77 (0.69, 0.83)	**2**	1.74 (1.18, 2.5)	**7**	1 (0.2, 3)	**7**	0.52 (0.4, 0.64)	**10**	1.75 (1.55, 1.98)	**2**	0.626	**5**		
(B) Second-line or later lines																	
CD8 + TILs	5	0.94 (0.79, 1)	**1**	0.47 (0.3, 0.65)	**12**	68.97 (3.04, 340.7)	**1**	4.06 (0.33, 13)	**3**	1.36 (1.13, 1.53)	**1**	0.92 (0.6, 1.3)	**12**	0.866	**1**		
ctDNA	6	0.88 (0.71, 0.97)	**2**	0.62 (0.43, 0.79)	**7**	18.79 (3.8, 62.54)	**2**	10.16 (0.71, 21)	**1**	1.27 (1.02, 1.48)	**2**	1.22 (0.84, 1.59)	**7**	0.813	**2**		
PLR	6	0.71 (0.5, 0.88)	**3**	0.58 (0.39, 0.76)	**8**	4.31 (1.21, 11.76)	**5**	3.19 (0.09, 13)	**4**	1.03 (0.71, 1.29)	**3**	1.14 (0.76, 1.51)	**8**	0.699	**6**		
NLR	8	0.71 (0.55, 0.83)	**4**	0.5 (0.38, 0.63)	**11**	2.73 (1.16, 5.52)	**8**	0.97 (0.08, 7)	**10**	1.02 (0.79, 1.23)	**4**	0.99 (0.74, 1.27)	**11**	0.639	**11**		
PD-L1 ≥ 1%	53	0.69 (0.63, 0.75)	**5**	0.51 (0.46, 0.56)	**10**	2.37 (1.71, 3.21)	**11**	0.61 (0.09, 2.33)	**11**	1 (1, 1)	**5**	1 (1, 1)	**10**	0.625	**12**		
irAEs	4	0.69 (0.45, 0.86)	**6**	0.68 (0.51, 0.83)	**5**	6.12 (1.72, 16.47)	**4**	6.98 (0.14, 17)	**2**	1 (0.66, 1.28)	**6**	1.35 (0.99, 1.67)	**5**	0.757	**3**		
LDH	5	0.65 (0.46, 0.82)	**7**	0.46 (0.32, 0.61)	**13**	1.85 (0.62, 4.42)	**12**	0.42 (0.05, 3)	**13**	0.94 (0.66, 1.2)	**7**	0.91 (0.62, 1.24)	**13**	0.682	**7**		
TMB	31	0.57 (0.48, 0.66)	**8**	0.69 (0.62, 0.75)	**4**	3.02 (1.91, 4.58)	**6**	2.22 (0.14, 9)	**5**	0.83 (0.69, 0.97)	**8**	1.35 (1.18, 1.53)	**4**	0.644	**10**		
PD-L1 ≥ 5%	18	0.56 (0.46, 0.66)	**9**	0.68 (0.6, 0.74)	**6**	2.75 (1.64, 4.26)	**7**	1.62 (0.14, 7)	**7**	0.81 (0.66, 0.97)	**9**	1.33 (1.16, 1.52)	**6**	0.666	**8**		
LIPI	3	0.52 (0.28, 0.74)	**10**	0.57 (0.37, 0.76)	**9**	1.69 (0.51, 4.27)	**13**	0.5 (0.05, 3.67)	**12**	0.75 (0.4, 1.09)	**10**	1.13 (0.73, 1.53)	**9**	0.586	**13**		
PD-L1 ≥ 10%	14	0.44 (0.33, 0.55)	**11**	0.76 (0.68, 0.82)	**3**	2.52 (1.4, 4.2)	**9**	1.59 (0.14, 5)	**8**	0.63 (0.48, 0.8)	**11**	1.49 (1.31, 1.68)	**3**	0.665	**9**		
PD-L1 ≥ 50%	4	0.36 (0.18, 0.58)	**12**	0.79 (0.65, 0.89)	**2**	2.47 (0.72, 6.39)	**10**	1.34 (0.11, 7)	**9**	0.52 (0.27, 0.83)	**12**	1.55 (1.27, 1.81)	**2**	0.705	**5**		
MSI	9	0.31 (0.2, 0.45)	**13**	0.93 (0.89, 0.96)	**1**	6.55 (2.68, 13.41)	**3**	2.04 (1, 5)	**6**	0.45 (0.28, 0.65)	**13**	1.83 (1.65, 2.03)	**1**	0.722	**4**		

Based on the network meta-analysis (NMA), the diagnostic efficacy (sensitivity, specificity, DOR, and other indicators and rankings) of immunotherapy biomarkers in first-line and second-line or later lines was compared. AUC, area under curve; CD8 + TILs, CD8-positive tumor-infiltrating lymphocytes; ctDNA, circulating tumor DNA; DOR, diagnostic odds ratio; irAEs, immune-related adverse events; LDH, lactate dehydrogenase; LIPI, Lung Immune Prognostic Index; MSI, microsatellite instability; NLR, neutrophil-to-lymphocyte ratio; PD-L1, programmed death-ligand 1; PLR, platelet-to-lymphocyte ratio; TMB, tumor mutational burden.

In the first-line setting, irAEs demonstrated the highest sensitivity (0.90, 95% CI: 0.83–0.95), while their specificity was relatively low (0.25, 95% CI: 0.16–0.38). ctDNA showed a strong overall performance, ranking second in sensitivity (0.80, 95% CI: 0.65–0.90) and first in DOR (6.73, 95% CI: 2.54–15.03) and superiority (7.96, 95% CI: 1–15). In contrast, PD-L1 ≥ 50% and PD-L1 ≥ 10% achieved the highest specificities (0.77, 95% CI: 0.72–0.82 and 0.77, 95% CI: 0.69–0.83, respectively), ranking first and second, but they had correspondingly low sensitivities (0.39 and 0.34, respectively). TMB offered a more balanced profile with moderate sensitivity (0.60, 95% CI: 0.52–0.67) and specificity (0.60, 95% CI: 0.53–0.67).

In the second or later-line settings, CD8+ TILs emerged as the most sensitive biomarker (0.94, 95% CI: 0.79–1.00) and also achieved the highest DOR (68.97, 95% CI: 3.04–340.70) and AUC (0.866). ctDNA continued to perform robustly, ranking second in sensitivity (0.88, 95% CI: 0.71–0.97), DOR (18.79, 95% CI: 3.80–62.54), and AUC (0.813). MSI was the standout marker for specificity (0.93, 95% CI: 0.89–0.96), ranking first, but it had the lowest sensitivity (0.31, 95% CI: 0.20–0.45) in this subgroup. irAEs demonstrated a notably higher specificity (0.68, 95% CI: 0.51–0.83) in later lines compared to first-line treatment, while maintaining good sensitivity (0.69, 95% CI: 0.45–0.86).

This analysis highlights that the diagnostic performance of biomarkers varies with the treatment line. Markers like CD8+ TILs and ctDNA show exceptional promise in the second-line or later setting, particularly for sensitivity. Conversely, the high specificity of PD-L1 at high cutoffs and MSI remains a consistent feature across lines, but their clinical utility as standalone tests may be limited by low sensitivity, especially in the first-line setting.

#### Subgroup analysis by biomarker methods

Given that the variability in detection methods is a major source of heterogeneity across studies, we performed a network meta-analysis to compare the diagnostic performance of different techniques within the same biomarker category. The comparative outcomes for the main biomarker are detailed in Table 5.

**Table 5:** Subgroup network meta-analysis by biomarker methods.

Marker methods	Number of studies	Sensitivity	RANK	Specificity	RANK	DOR	RANK	Superiority	RANK	Relative Sensitivity	RANK	Relative Specificity	RANK	AUC	RANK	
(A) PD-L1																
SP263	15	0.64 (0.51, 0.76)	**1**	0.59 (0.49, 0.69)	**3**	2.69 (1.79, 3.88)	**1**	3.06 (0.33, 7)	**1**	1.04 (0.82, 1.26)	**1**	1.07 (0.87, 1.29)	**3**	0.662	**1**	
Dako 22C3	76	0.62 (0.57, 0.67)	**2**	0.55 (0.51, 0.59)	**4**	2 (1.72, 2.3)	**3**	0.93 (0.2, 3)	**4**	1 (1, 1)	**2**	1 (1, 1)	**4**	0.624	**3**	
SP142	40	0.61 (0.52, 0.68)	**3**	0.6 (0.54, 0.67)	**2**	2.35 (1.87, 2.92)	**2**	2.28 (0.33, 5)	**2**	0.98 (0.83, 1.15)	**3**	1.09 (0.95, 1.25)	**2**	0.64	**2**	
Dako 73-10	5	0.58 (0.36, 0.79)	**4**	0.51 (0.32, 0.69)	**5**	1.61 (0.76, 3.14)	**5**	0.64 (0.11, 3)	**5**	0.95 (0.58, 1.31)	**4**	0.93 (0.58, 1.28)	**5**	0.576	**5**	
Dako 28-8	44	0.47 (0.4, 0.56)	**5**	0.68 (0.62, 0.73)	**1**	1.92 (1.56, 2.35)	**4**	1.26 (0.33, 3)	**3**	0.77 (0.63, 0.92)	**5**	1.23 (1.08, 1.38)	**1**	0.599	**4**	
(B) TMB																
WES	19	0.59 (0.49, 0.68)	**1**	0.67 (0.57, 0.7)	**2**	2.98 (1.92, 4.53)	**1**	1.25 (0.33, 3)	**1**	1.15 (0.92, 1.39)	**1**	0.93 (0.79, 1.08)	**2**	0.639	**1**	
Large Panel	47	0.52 (0.45, 0.58)	**2**	0.72 (0.66, 0.73)	**1**	2.72 (2.07, 3.56)	**2**	1.01 (0.33, 3)	**2**	1 (1, 1)	**2**	1 (1, 1)	**1**	0.629	**2**	
(C) ctDNA																
Medium Panel	3	0.8 (0.51, 0.96)	**1**	0.66 (0.45, 0.82)	**3**	14.11 (1.8, 57.67)	**2**	1.56 (0.2, 5)	**2**	1.16 (0.7, 1.78)	**1**	0.95 (0.63, 1.3)	**3**	0.822	**1**	
Small Panel	3	0.8 (0.5, 0.95)	**2**	0.69 (0.51, 0.84)	**2**	15.1 (2.16, 55.46)	**1**	2.13 (0.2, 5)	**1**	1.15 (0.7, 1.71)	**2**	1.01 (0.71, 1.37)	**1**	0.702	**3**	
Large Panel	7	0.71 (0.48, 0.88)	**3**	0.7 (0.55, 0.81)	**1**	7.21 (1.83, 19.59)	**3**	1.15 (0.2, 5)	**3**	1 (1, 1)	**3**	1 (1, 1)	**2**	0.714	**2**	
(D) MSI																
IHC	4	0.52 (0.3, 0.75)	**1**	0.78 (0.53, 0.93)	**2**	5.94 (0.96, 20.7)	**2**	1.08 (0.33, 3)	**1**	1 (1, 1)	**1**	1 (1, 1)	**2**	0.727	**1**	
NGS	8	0.25 (0.14, 0.43)	**2**	0.92 (0.81, 0.98)	**1**	5.96 (1.14, 17.76)	**1**	1.03 (0.33, 3)	**2**	0.51 (0.24, 1.05)	**2**	1.2 (0.97, 1.72)	**1**	0.695	**2**	
(E) CD8 + TILs																
Clone 4B11	7	0.77 (0.47, 0.95)	**1**	0.53 (0.28, 0.76)	**1**	7.06 (0.76, 27.85)	**1**	1.79 (0.33, 3)	**1**	1 (1, 1)	**1**	1 (1, 1)	**1**	0.718	**1**	
Clone C8/144B	3	0.68 (0.45, 0.88)	**2**	0.49 (0.3, 0.67)	**2**	2.72 (0.59, 7.98)	**2**	0.93 (0.33, 3)	**2**	0.92 (0.55, 1.54)	**2**	0.99 (0.5, 1.89)	**2**	0.54	**2**	

This network meta-analysis (NMA) compares the diagnostic performance of different detection methods within the same biomarker category. For each biomarker group, the methods are ranked (RANK 1 being the best) based on their performance in sensitivity, specificity, diagnostic odds ratio, and other comparative indicators. AUC, area under the curve; CD8+ TILs, CD8-positive tumor-infiltrating lymphocytes; ctDNA, circulating tumor DNA; DOR, diagnostic odds ratio; IHC, immunohistochemistry; MSI, microsatellite instability; NGS, next-generation sequencing; PD-L1, programmed death-ligand 1; TMB, tumor mutational burden; WES, whole-exome sequencing.

Among the five PD-L1 detection assays, SP263 demonstrated the highest sensitivity (0.64, 95% CI: 0.51–0.76) and the highest DOR (2.69, 95% CI: 1.79–3.88), ranking first in both metrics. The Dako 28-8 assay showed the highest specificity (0.68, 95% CI: 0.62–0.73). Conversely, the Dako 73-10 assay consistently demonstrated the lowest performance, ranking fifth in specificity (0.51, 95% CI: 0.32–0.69), DOR (1.61, 95% CI: 0.76–3.14), and AUC (0.576).

For TMB assessment, whole-exome sequencing (WES) was compared with large panel approaches based on the genomic coverage of the testing methods, to better perform subgroup analysis. WES outperformed the Large Panel in several key metrics, ranking first in sensitivity (0.59, 95% CI: 0.49–0.68), DOR (2.98, 95% CI: 1.92–4.53), and AUC (0.639). However, the large panel method showed superior specificity (0.72, 95% CI: 0.66–0.73).

Among ctDNA detection panels, methods were categorized into large, medium, and small panels according to the breadth of gene coverage, allowing a structured subgroup comparison. The medium panel achieved the highest sensitivity (0.80, 95% CI: 0.51–0.96) and the highest AUC (0.822), ranking first in both. The small panel showed the highest DOR (15.1, 95% CI: 2.16–55.46), ranking first for this metric. The large panel demonstrated the highest specificity (0.70, 95% CI: 0.55–0.81), ranking first in this category.

For MSI detection, immunohistochemistry (IHC) demonstrated higher sensitivity (0.52, 95% CI: 0.30–0.75) and a superior AUC (0.727), ranking first in both. In contrast, next-generation sequencing (NGS) showed markedly higher specificity (0.92, 95% CI: 0.81–0.98) and a slightly higher DOR (5.96, 95% CI: 1.14–17.76), ranking first in these two metrics, albeit with a much lower sensitivity (0.25, 95% CI: 0.14–0.43).

Between the two CD8+ TIL clones analyzed, Clone 4B11 consistently outperformed Clone C8/144B. Clone 4B11 ranked first in sensitivity (0.77, 95% CI: 0.47–0.95), DOR (7.06, 95% CI: 0.76–27.85), and AUC (0.718). While both clones showed modest specificity, Clone 4B11 had a slightly higher value (0.53, 95% CI: 0.28–0.76) and ranked first.

In terms of heterogeneity (see Supplemental Digital Content Material 6.1–6.5, available at: http://links.lww.com/JS9/G519), for TMB, despite high heterogeneity in specificity for both large panel and WES (I^2^ > 80%), WES showed low heterogeneity in sensitivity and DOR (I^2^ < 50%). In ctDNA, the large panel had consistently low heterogeneity across all metrics (I^2^ < 50%). Similarly, the Clone C8/144B for CD8 + TILs and IHC for MSI also exhibited low heterogeneity. Expectedly, substantial heterogeneity was observed in PD-L1 assay groups for sensitivity and specificity, due to differences in scoring thresholds and platforms. To conclude, this subgroup network meta-analysis identified leading detection methods for each biomarker (e.g., SP263 for PD-L1, WES for TMB) and revealed that performance is highly metric-dependent. The findings directly address a key source of heterogeneity across studies and emphasize the need for methodological standardization in biomarker research to facilitate valid cross-trial comparisons.

## Discussion

Cancer immunotherapy has fundamentally transformed treatment paradigms across diverse malignancies. This expansion now encompasses neoadjuvant and adjuvant settings, demonstrating benefit for early/locally advanced stage patients^[208]^. Nevertheless, immunotherapy responses display substantial interpatient heterogeneity, governed by a complex interplay of tumor-intrinsic factors (e.g., mutational landscape), host immunity (notably T-cell repertoire diversity), and treatment-specific parameters^[46,209,210]^. This biological complexity necessitates multidimensional biomarker strategies to identify optimal candidates, thereby maximizing therapeutic efficacy while mitigating the risk of serious immune-related adverse events.

This comprehensive network meta-analysis provides a rigorous comparative evaluation of 13 immunotherapy biomarkers across advanced solid tumors. Our analysis revealed significant changes in biomarker performance characteristics, emphasizing several important clinical implications, especially regarding the selection of precision biomarkers. ctDNA demonstrated exceptional overall discriminative ability, achieving the highest sensitivity (0.82, 95% CI: 0.72–0.89), DOR (9.75, 95% CI: 5.20–16.73), and AUC (0.769) among all biomarkers. Its performance was particularly notable in NSCLC, where it maintained high sensitivity (0.77, 95% CI: 0.63–0.88) and DOR (9.63, 95% CI: 3.93–21.14). PD-L1 expression exhibited clear threshold-dependent characteristics, with higher cutoffs (≥50%) achieving superior specificity (0.78, 95% CI: 0.73–0.81) at the cost of sensitivity (0.42, 95% CI: 0.36–0.49). CD8+ TILs emerged as a robust predictor in NSCLC, ranking second in DOR (6.04, 95% CI: 2.12–14.54), while MSI status demonstrated exceptional performance in GI cancers with the highest specificity (0.96, 95% CI: 0.93–0.99) and DOR (15.53, 95% CI: 4.05–43.89). Among inflammatory markers, PLR showed promising performance in chemo-immunotherapy combinations with the highest AUC (0.794), while NLR demonstrated more modest discriminative capacity (DOR: 1.86, 95% CI: 1.24–2.73). Importantly, subgroup analyses also revealed that biomarker performance varied substantially across treatment settings and lines, with ctDNA and CD8+ TILs showing exceptional promise in later-line treatments, while PD-L1 high cutoffs and MSI maintained consistent specificity across contexts. These findings underscore the critical importance of context-specific biomarker selection based on cancer type, treatment regimen, and line of therapy.

Critically, these findings not only corroborate but also extend conclusions from prior research. Regarding ctDNA, our analysis confirms its superior sensitivity, aligning with studies emphasizing its dynamic monitoring utility. This noninvasive modality enables real-time tracking of tumor clonal evolution and treatment response^[211]^. A meta-analysis of 18 studies demonstrated that ctDNA reduction 6-16 weeks posttreatment initiation significantly correlated with improved PFS (HR 0.20, 95% CI 0.14–0.28, *P* < 0.001)^[212]^. While their work zeroed in on the specific prognostic significance of dynamic changes in ctDNA for PFS and OS, our NMA offers a more expansive, comparative assessment of ctDNA in relation to 12 other predictive biomarkers across a wide array of cancer types and clinical scenarios. Furthermore, a study integrated in our network meta-analysis revealed that patients achieving ctDNA clearance after immunotherapy exhibited significantly higher clinical response rates (75.0% vs. 0.0% without clearance)^[17]^. Notably, ctDNA detects treatment response earlier than radiographic imaging. In neoadjuvant chemotherapy cohorts, ctDNA clearance at cycles 2–3 predicted subsequent pathological complete response (pCR), whereas imaging assessment required extended timelines^[213,214]^. However, the clinical application of ctDNA remains challenged by methodological heterogeneity and unresolved practical considerations. As our meta-regression analysis of heterogeneity sources confirms (Supplemental Digital Content Fig. S2.7, available at: http://links.lww.com/JS9/G519), factors such as the detection method (accounting for 22.7% of sensitivity variance) and expected ctDNA response classification (contributing to 29.6% of sensitivity and 12.2% of specificity variance) are significant sources of variability. This lack of harmonization means that a “positive” or “negative” result is highly assay-dependent, raising the critical risk of false-positive readings and potential downstream overtreatment of patients who might not truly benefit. Furthermore, key barriers to routine clinical implementation, such as the cost-effectiveness and turnaround time for ctDNA NGS, remain inadequately addressed in current literature. Notwithstanding these practical considerations, our findings undeniably affirm the superior predictive performance of ctDNA and its critical value in complementing traditional static biomarkers. Standardizing assay protocols, validating in trials, and conducting health-economic analyses are key to unlocking ctDNA’s potential for personalizing cancer immunotherapy.

Conversely, PD-L1 expression assessed by IHC remains the most validated biomarker for predicting ICI response^[215]^. Its predictive value is well-established: high PD-L1 expression (e.g., TPS ≥ 50%) correlates with superior outcomes in first-line pembrolizumab monotherapy^[216]^. In the KEYNOTE-052 urothelial carcinoma trial, ORR were significantly higher in patients with PD-L1 expression ≥10% versus <1% (39% vs. 11%)^[217]^. Our network meta-analysis further refines this understanding, revealing substantial performance heterogeneity across different PD-L1 assays. Notably, the SP263 assay demonstrated the highest sensitivity and DOR, whereas the 73-10 assay consistently ranked lowest across all performance metrics. This observed variability is strongly supported by our meta-regression, which identified the detection method as a significant source of variation, explaining up to 24.5% of the variance in sensitivity and 20.4% in specificity for PD-L1 ≥ 10% thresholds (Supplemental Digital Content Fig. S2.3, available at: http://links.lww.com/JS9/G519). While the Blueprint project demonstrated analytical concordance across some antibodies (22C3, 28-8, SP263)^[218]^, our data indicate that threshold- and assay-specific performance differences persist in clinical practice, complicating cross-study comparisons and routine application. Our heterogeneity analysis further uncovers that scoring type, such as tumor-positive score and combined positive score, was another notable factor, accounting for up to 13.4% of specificity variance at the PD-L1 ≥ 1% threshold (Supplemental Digital Content Fig. S2.1, available at: http://links.lww.com/JS9/G519). Spatial and temporal heterogeneity further compromises the reliability of single-time-point assessments. Consequently, optimization of this complex biomarker for clinical application remains imperative. Moreover, spatial and temporal heterogeneity further compromises the reliability of single-time-point assessments. Consequently, optimization of this complex biomarker for clinical application remains imperative.

MSI-H/dMMR represents a highly predictive pan-cancer biomarker for immunotherapy response^[219]^. Clinical trials (e.g., KEYNOTE-164) validate the efficacy and safety of pembrolizumab in MSI-H/dMMR colorectal cancer, attributable to its mechanistic role in driving ICI responsiveness^[220,221]^, establishing the first tissue-agnostic immunotherapy indication^[222]^. However, its clinical application is constrained by several layers of limitations. First, our network meta-analysis quantifies its fundamental diagnostic profile: high specificity (0.89, 95% CI: 0.85–0.93) but notably suboptimal sensitivity (0.36, 95% CI: 0.27–0.46), inherently limiting its ability to identify all potential responders. Second, this performance is further confounded by methodological heterogeneity (Supplemental Digital Content Fig. S2.6, available at: http://links.lww.com/JS9/G519). Our analysis identifies the detection method (IHC vs. NGS) as a major source of variability, explaining over half of the variance in sensitivity (51.8%). Finally, beyond these assay-related issues, multiple tumor-intrinsic factors contribute to clinical resistance, including primary resistance in >50% of dMMR colorectal cancer patients^[7]^, immune evasion via HLA loss^[223]^, spatial heterogeneity^[224–226]^, and the low prevalence in common solid tumors^[227]^. Strategies to overcome these limitations include combining MSI status with TMB, immune gene signatures, or TME profiling, supplemented by functional neoantigen-specific T-cell assays.

TMB quantifies somatic nonsynonymous mutations within a defined genomic region, reflecting tumor neoantigen burden and predicting immunotherapy response across malignancies^[46,228]^. Following KEYNOTE-158 results demonstrating superior response in high-TMB patients^[194]^, the FDA approved TMB as the first tissue-agnostic companion diagnostic for pembrolizumab in 2020. CheckMate-227 further established the predictive value of TMB in NSCLC, where nivolumab/ipilimumab outperformed chemotherapy irrespective of PD-L1 status^[229]^. Our NMA indicates the balanced diagnostic profile of TMB: moderate sensitivity (0.56, 95% CI: 0.5–0.6) and superior specificity (0.69, 95% CI: 0.65–0.73). However, methodological heterogeneity significantly compromises cross-study comparability and predictive accuracy. Our meta-regression quantifies key sources of this variation, identifying that the cutoff method explains 7.9% of the variance in specificity (*P* < 0.05), while the detection platform and treatment type also contribute notably to sensitivity heterogeneity (Supplemental Digital Content Fig. S2.5, available at: http://links.lww.com/JS9/G519). This underscores the pressing need for standardized TMB assessment protocols to harmonize variable approaches across tissue/liquid biopsies, detection platforms, analytical pipelines, and threshold determinations.

The tumor immune landscape, particularly TILs, critically predicts immunotherapy response. CD8+ TIL density correlates with improved PFS/ORR^[162,173]^. In our analysis, high CD8+ TIL intensity demonstrated good sensitivity (0.69, 95% CI: 0.58–0.79) but moderate specificity (0.59; 95% CI: 0.49–0.67) in predicting treatment outcome. This predictive performance varied substantially across cancer subtypes, a variation primarily driven by how distinct tumor microenvironments (TMEs) shape the hierarchical architecture of CD8+ T cell exhaustion. In immunogenic tumors like NSCLC and melanoma, the TME often maintains niches that support TCF1^+^ progenitor exhausted T cells, which retain stem-like self-renewal capacity and drive the proliferative burst following PD-1/PD-L1 blockade^[230]^. This context makes preexisting CD8+ T-cell infiltration a strong predictor of response to immunotherapy. In contrast, gastrointestinal cancers exhibit strong immunosuppressive signals, such as abundant Tregs, tumor-associated macrophages, and high TGF-β, that push TCF1^+^ progenitors toward terminal exhaustion, rendering them poorly responsive to current checkpoint inhibitors^[231]^. Furthermore, the effector-to-regulatory T cell ratio (e.g., CD8 +/Treg) modulates antitumor immunity, while complementary biomarkers such as immune checkpoint expression (PD-L1, LAG-3), myeloid polarization (M1/M2 ratio), and circulating TCR clonality offer additional resolution^[227,232]^.

Regarding inflammatory indices, the NLR reflects systemic inflammation and immune balance. Baseline or posttreatment NLR elevation (typically ≥ 2.34 or ≥ 5) correlates with poor outcomes. In ICI-treated patients (*n* > 2000), high NLR predicted reduced OS (HR = 2.17; 95% CI: 1.89–2.50) and lower response rates (18% vs. 29%)^[199]^. Early ΔNLR monitoring (AUC = 0.706) outperforms baseline assessment (AUC = 0.600) in NSCLC^[233,234]^. PLR integrates protumor thrombotic effects and anti-tumor lymphocyte activity^[235–237]^, with low PLR consistently associating with improved OS/ORR across tumors^[20,193]^. PLR demonstrates superior specificity versus NLR despite comparable sensitivity. LDH, reflecting tumor metabolic activity and hypoxia, shows an inverse association with ICI response; combining with TMB improves prediction in melanoma/HCC^[238,239]^. Our analysis reveals the limited predictive performance of LDH, which, despite a moderate sensitivity of 0.66 (95% CI: 0.55–0.76, rank 5), showed the lowest specificity among all biomarkers at 0.40 (95% CI: 0.31–0.49, rank 13). The LIPI, which integrates LDH and NLR, also demonstrated suboptimal utility with a DOR of 1.65 (95% CI: 1.01–2.54, rank 12). Notably, in the HNSCC subgroup, LDH showed the highest predictive sensitivity (0.74, 95% CI: 0.44–0.94). This may be due to HNSCC’s highly glycolytic and metabolically dysregulated tumor microenvironment, correlating more strongly with treatment response^[240]^.

Ultimately, irAEs potentially reflect antitumor T-cell activation. Advanced NSCLC patients developing irAEs on nivolumab showed higher ORR (37% vs. 17%)^[35]^. Phase 3 data reveal grade 1–2 irAEs correlate with improved OS (HR = 0.69), though grade ≥3 events may negate the benefit due to treatment discontinuation. Organ-specific patterns exist: cutaneous/endocrine/GI irAEs associate with better outcomes, while pulmonary/hepatobiliary toxicities may offset efficacy^[64]^. Our NMA supports irAEs monitoring with a sensitivity of 0.69 (95% CI: 0.6–0.77), though predictive value varies by type, grade, and therapeutic agent. However, its predictive performance exhibits substantial heterogeneity (Supplemental Digital Content Fig. S2.13, available at: http://links.lww.com/JS9/G519), primarily driven by treatment type (R^2^ = 52.3% for sensitivity, 79.0% for specificity, *P* < 0.01), suggesting that the immunomodulatory mechanism of different therapeutic agents significantly influences irAEs-based prediction.

Another key finding of our study is the generalizability of biomarker predictive performance across different treatment modalities. While the aggregate network meta-analysis (Table 1) provides a consolidated overview, the subgroup analyses (Table 3) show that predictive efficacy depends on treatment context. For instance, TMB has higher sensitivity in dual immunotherapy than in monotherapy, suggesting its predictive power is enhanced by combined immune checkpoint inhibition. In contrast, ctDNA performs consistently across monotherapy and chemo-immunotherapy, highlighting its robustness as a more agnostic biomarker. These results indicate that biomarker selection must consider the intended therapeutic regimen. Our data support a shift toward context-specific biomarker validation.

Methodologically, placing our findings within the context of recent meta-analyses, this NMA provides a unified comparative assessment of 13 biomarkers that extends beyond prior focused investigations. Our analysis confirms established findings, including the moderate performance of PD-L1 and TMB reported by Lu *et al* and the predictive value of irAEs demonstrated by Zhou *et al*^[241,242]^, while quantitatively situating these markers within a broader evaluative framework. Notably, we identify ctDNA as the leading predictor, moving beyond its previously recognized role in treatment monitoring. Our study also offers a crucial clarification regarding inflammatory biomarkers: while LIPI shows strong prognostic value for survival stratification, as confirmed by Guo *et al*^[243]^, our comparative approach reveals its more limited utility for predicting initial treatment response. Furthermore, in extending the work of Shi *et al*^[244]^, whose network meta-analysis of seven biomarkers highlighted multiplex IHC as the most sensitive, our broader panel and context-aware subgroup analyses provide a more granular and clinically actionable biomarker hierarchy to advance personalized immunotherapy decision-making.

### Limitations

Methodological limitations merit consideration. First, significant heterogeneity (I^2^ > 90%) was observed, primarily stemming from variations in biomarker assays, divergent detection methods, thresholds, cut-off derivation, differences in tumor subtypes, and divergent treatment settings. This high degree of heterogeneity, while partially addressed through meta-regression, underscores that the pooled estimates should be interpreted as an average across diverse methodologies and populations, potentially compromising the reliability and robustness of specific comparative conclusions. Second, the retrospective design of most included studies carries inherent risks of selection and information bias, necessitating prospective validation for definitive clinical utility assessment. Third, our subgroup analyses were constrained by limited data for specific cancer types. The underrepresentation of cohorts such as urothelial carcinoma and the scarcity of data for promising biomarkers like ctDNA within gastrointestinal and urothelial cancers likely result in underpowered subgroup conclusions, limiting our ability to discern true cancer-specific predictive patterns.

Finally, publication bias is a pertinent concern. Deeks’ funnel plot asymmetry tests indicated no significant bias for several biomarkers (e.g., CD8+ TILs, ctDNA, TMB). However, significant asymmetry was detected for others, including MSI, irAEs, and PD-L1 ≥ 50%. This suggests that the literature on these markers may be skewed toward positive results. Consequently, the exceptionally high specificity observed for MSI and the efficacy estimates for PD-L1 ≥ 50% might be inflated, as studies confirming their predictive value are more likely to be published than those with null findings. This potential bias necessitates cautious interpretation of their performance and tempers the strength of their clinical recommendation, highlighting the need for validation in broader, unselected patient cohorts.

While ORR remains valuable, its imperfect OS correlation in immunotherapy due to delayed survival curve separation, postprogression interventions, and pseudoprogression requires acknowledgment. Standardized survival dichotomization approaches are lacking: median OS cutoffs invite right-censoring bias, while landmark analyses demand large samples and exhibit timepoint sensitivity.

Inclusion of single-biomarker studies introduced network imbalances (isolated nodes distorting effect estimates), amplified heterogeneity (masking true effects vs. methodological variations), and compromised transitivity. Sensitivity analysis (see Supplemental Digital Content Table S3, available at: http://links.lww.com/JS9/G519) confirmed robustness for ctDNA/MSI/PD-L1 ≥ 50% but revealed instability in inflammatory markers (NLR/PLR/LDH), warranting cautious application. Retaining such studies prevented selection bias but underscores the need for standardized multimarker protocols and enhanced NMA frameworks.

### Clinical implications and recommendations

This comprehensive network meta-analysis provides a robust evidence base for refining biomarker-guided immunotherapy strategies. To enhance the accessibility of this complex biomarker landscape, a glossary of key terms is provided in the Supplemental Digital Content Table S4, available at: http://links.lww.com/JS9/G519. To bridge current evidence gaps, particularly the scarcity of data for potent biomarkers like ctDNA in specific GI cancers, HNSCC, and urothelial cancers, we urgently recommend targeted prospective trials to validate their utility in these underrepresented populations. For immediate clinical translation, we propose a structured, step-by-step framework: initial screening should utilize ctDNA for its superior sensitivity, effectively ruling out nonresponders, followed by confirmatory testing with either MSI or PD-L1 (≥50%) to ensure treatment specificity, particularly in NSCLC. This binary approach ensures both comprehensive patient capture and precision in therapeutic assignment.

Building upon this foundational strategy, clinical implementation should incorporate context-aware biomarker stratification. Treatment algorithms should be refined by integrating cancer-specific biomarkers, such as CD8+ TILs in NSCLC or TMB in melanoma, and regimen-specific markers, like PD-L1 (≥5%) for immunotherapy-targeted combinations. Furthermore, dynamic monitoring through serial ctDNA and TMB assessment is strongly advised to enable real-time efficacy evaluation and early resistance detection. This rationale naturally extends to developing integrated biomarker panels, combining the high sensitivity of ctDNA with the high specificity of MSI or PD-L1, thereby creating a more robust predictive system than any single marker alone.

Looking ahead, the field must prioritize next-generation biomarker development. Future efforts should focus on constructing machine learning models that integrate TMB, PD-L1, and novel markers, implementing comprehensive dynamic monitoring via liquid biopsy and functional imaging, and systematically evaluating emerging candidates like the gut microbiome and spatial transcriptomics in biomarker-driven trials. Through this multifaceted approach, bridging immediate clinical application with forward-looking research, we can advance toward truly personalized immunotherapy.

## Conclusions

This network meta-analysis of 13 immunotherapy biomarkers identifies ctDNA as the top-performing predictor across multiple cancer types, achieving the highest rankings in sensitivity, DOR, and superiority. CD8+ TILs also show consistent predictive value, while TMB exhibits strong specificity, particularly in NSCLC and melanoma. PD-L1 displays threshold-dependent accuracy (elevated cutoffs enhance specificity), while MSI status serves as a high-specificity confirmatory marker. Performance varies by the cancer type, therapeutic modality (monotherapy or combination therapy), and the treatment line. These findings advocate for personalized biomarker-guided strategies, necessitating multimodal integration with tumor context. Future research must address heterogeneity/standardization to refine precision immunotherapy patient selection.

## Data Availability

All data generated in this study are included in the manuscript and supplementary materials. Additional information is available from the corresponding author. Further inquiries can be directed to the corresponding author.

## Research registration unique identifying number (UIN)

This study was registered with PROSPERO (registration number CRD42023472497).
